# Attitudes of European students towards family decision-making and the harmonisation of consent systems in deceased organ donation: a cross-national survey

**DOI:** 10.1186/s12889-022-14476-z

**Published:** 2022-11-15

**Authors:** Alberto Molina-Pérez, Gabriele Werner-Felmayer, Kristof Van Assche, Anja M. B. Jensen, Janet Delgado, Magdalena Flatscher-Thöni, Ivar R. Hannikainen, David Rodriguez-Arias, Silke Schicktanz, Sabine Wöhlke

**Affiliations:** 1 Instituto de Estudios Sociales Avanzados (IESA), CSIC, Plaza Campo Santo de los Mártires 7, 14004 Córdoba, Spain; 2 FiloLab–UGR, Philosophy I Department, University of Granada, Granada, Spain; 3 Public Issues Working Group, ELPAT-ESOT, Padova, Italy; 4 Institute of Biological Chemistry, Biocentre, and Bioethics Network Ethucation, Medical University Innsbruck, Innsbruck, Austria; 5 Research Group Personal Rights and Property Rights, Faculty of Law, University of Antwerp, Antwerp, Belgium; 6 Section for Health Services Research, Department of Public Health, University of Copenhagen, Copenhagen, Denmark; 7 Department of Public Health, Health Services Research and Health Technology Assessment, UMIT – Private University for Health Sciences, Medical Informatics and Technology, Hall in Tyrol, Austria; 8 Department of Medical Ethics and History of Medicine, University Medical Centre Göttingen, Göttingen, Germany; 9 Department of Health Science, Hamburg University of Applied Science, Hamburg, Germany

**Keywords:** Organ donation, Consent, Family involvement, Policy harmonisation, Survey

## Abstract

**Background:**

European countries are increasingly harmonising their organ donation and transplantation policies. Although a growing number of nations are moving to presumed consent to deceased organ donation, no attempts have been made to harmonise policies on individual consent and the role of the family in the decision-making process. Little is known about public awareness of and attitudes towards the role of the family in their own country and European harmonisation on these health policy dimensions. To improve understanding of these issues, we examined what university students think about the role of the family in decision-making in deceased organ donation and about harmonising consent policies within Europe.

**Methods:**

Using *LimeSurvey*© software, we conducted a comparative cross-sectional international survey of 2193 university students of health sciences and humanities/social sciences from Austria (339), Belgium (439), Denmark (230), Germany (424), Greece (159), Romania (190), Slovenia (190), and Spain (222).

**Results:**

Participants from opt-in countries may have a better awareness of the family’s legal role than those from opt-out countries. Most respondents opposed the family veto, but they were more ambivalent towards the role of the family as a surrogate decision-maker. The majority of participants were satisfied with the family’s legal role. However, those who were unsatisfied preferred to limit family involvement. Overall, participants were opposed to the idea of national sovereignty over consent policies. They favoured an opt-out policy harmonisation and were divided over opt-in. Their views on harmonisation of family involvement were consistent with their personal preferences.

**Conclusions:**

There is overall division on whether families should have a surrogate role, and substantial opposition to granting them sole authority over decision-making. If European countries were to harmonise their policies on consent for organ donation, an opt-out system that grants families a surrogate decision-making role may enjoy the widest public support.

**Supplementary Information:**

The online version contains supplementary material available at 10.1186/s12889-022-14476-z.

## Background

Organ transplantation numbers have steadily risen, amounting to 153,863 solid organs transplanted worldwide in 2019 [1]. Yet, this increase cannot meet the growing demand. In the European Union (EU), 34,285 transplants were performed in 2019, but 54,383 patients were still on a waiting list at the end of that year [2]. Donation rates further decreased during the first year of the Covid-19 pandemic (worldwide by about 18%; 28,212 transplants in EU) (EDQM, 2021). The average number of people who died per day while waiting for a transplant in the EU rose from 9 to 10 in 2019 [2] to 11 in 2020 [3].

To increase donation rates, European transplant organisations have collaborated over the past decades in improving deceased organ donation programmes through reciprocal learning, training, and benchmarking. Following the EU Action Plan on Organ Donation and Transplantation (2009–2015) and initiatives by the Council of Europe, EU member states have also progressed towards harmonisation of transplant practice and regulations [4]. Overall, EU member states have jointly increased the annual number of transplants from 28,110 in 2008 to 32,707 in 2015 (+ 16.4%) [5]. Nevertheless, major variations in deceased organ donation rates persist, ranging from 4.6 donors per million population (PMP) in Greece and even less in Romania (3.4) and Bulgaria (0.6), to more than 25 donors PMP in Croatia, Portugal, and Spain [3]. These disparities mean that some EU patients experience longer waiting lists and a higher risk of dying before transplantation.

Several factors may contribute to these disparities, including organisational measures (e.g., availability and role of transplant coordinators), mortality rates from causes that may well result in brain death (e.g., strokes and road accidents), and variations in clinical practice in donation after circulatory death (e.g., in duration of no-touch periods) as outlined by Lomero et al. [6]. Other factors involve the ethical and social aspects of organ donation and transplantation such as ambivalence towards brain death criteria, differences in how families make sense of donation decisions, emotional attitudes regarding the dead body, public acceptability of deceased donation, and levels of trust in and solidarity with the healthcare system [7–11]. In addition, and highly relevant for our study, there are distinct ways of valuing autonomy and family relationships between European regions, for example with regard to the authority of the family to overrule the deceased’s last wishes [12, 13]. It is uncertain whether and, if so, to what extent regional variations in valuing autonomy and family relationships constitute a challenge for introducing regulatory harmonisation on organ donation and to which degree they affect national policies with regard to individual consent and the role of the family in organ donation.

Public awareness of the consent model in place and the role relatives play in the decision-making process is also crucial for the autonomy of citizens’ decisions whether to donate or not [14, 15]. A systematic review showed that people in opt-out (i.e., presumed consent) countries have less awareness of their national consent policy than those living in opt-in (i.e., explicit consent) countries [16]. Furthermore, it is suggested that Europeans tend to prefer opt-in and mandatory choice policies rather than opt-out [16]. Both findings are worrying as individuals who neither wish to become organ donors nor support presumed consent policies may be unwittingly treated as potential donors [15]. In addition, this review suggested that, overall, only a minority of the public is aware of the role of the family in their country [17]. However, because of the scarcity and heterogeneity of data sources, the review pleaded for comparative research in a broader group of countries based on a common conceptual framework and properly standardised questionnaires.

Following this recommendation, we conducted a cross-national survey on university students’ awareness of and attitudes towards organ donation in Austria, Belgium, Denmark, Germany, Greece, Romania, Slovenia, and Spain. A recent analysis based on our survey’s data about the consent systems identified quality indicators for organ removal policies and showed that citizens’ support for the consent policy is crucial from the perspective of good policy governance in democratic societies [15]. Here, we focussed on the survey’s results regarding university students’ attitudes towards the role of families in deceased organ donation. The role families play in the consent process in deceased organ donation is highly complex. It consists of different approaches that are situated on a continuum from having no role at all, to being just a witness or also a surrogate decision-maker, to having full authority to make decisions regardless of the deceased’s wishes. These approaches have recently been conceptualised in a framework developed by Delgado and colleagues that describes four levels of family involvement [18]. Here, we analysed and interpreted our survey’s results on the basis of that framework.

As attitudes towards family involvement in decision-making are linked to acceptance or rejection of regulatory attempts at policy harmonisation, we also analysed how university students view the possible harmonisation of consent models within Europe. Understanding what university students believe about the role of families in decision-making in deceased organ donation as well as about harmonising consent policies can provide relevant knowledge to improve public education and communication strategies. Students, as well as being capable of issuing -reasoned opinion about complex issues are at a crucial age in the development of moral reasoning [19] and the formation of political attitudes [20] In this regard, their views on policy harmonisation are of particular interest, insofar as they are likely to heavily inform policy development in the following decades.

## Methods

### Survey questionnaire and study sample

**Fig. 1:**
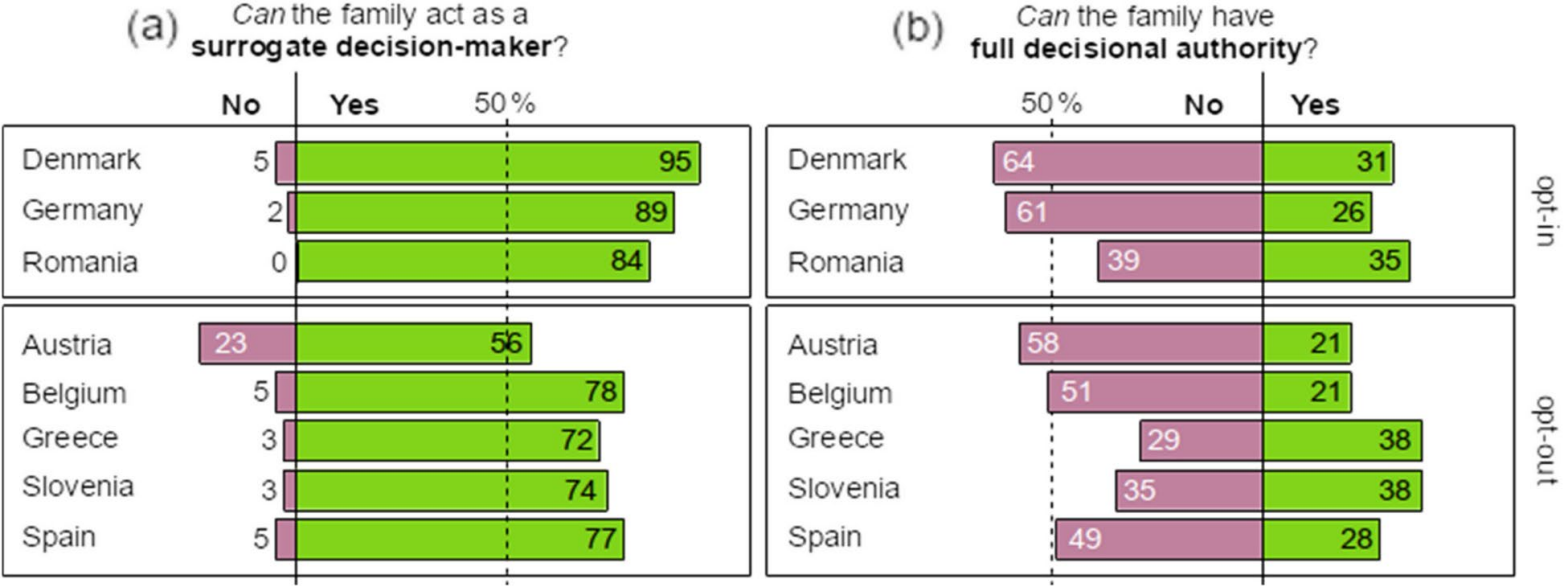
Respondents’ awareness of the role of the family. Percentages do not add up to 100 due to the omission of ‘Don’t know’ responses

**Fig. 2:**
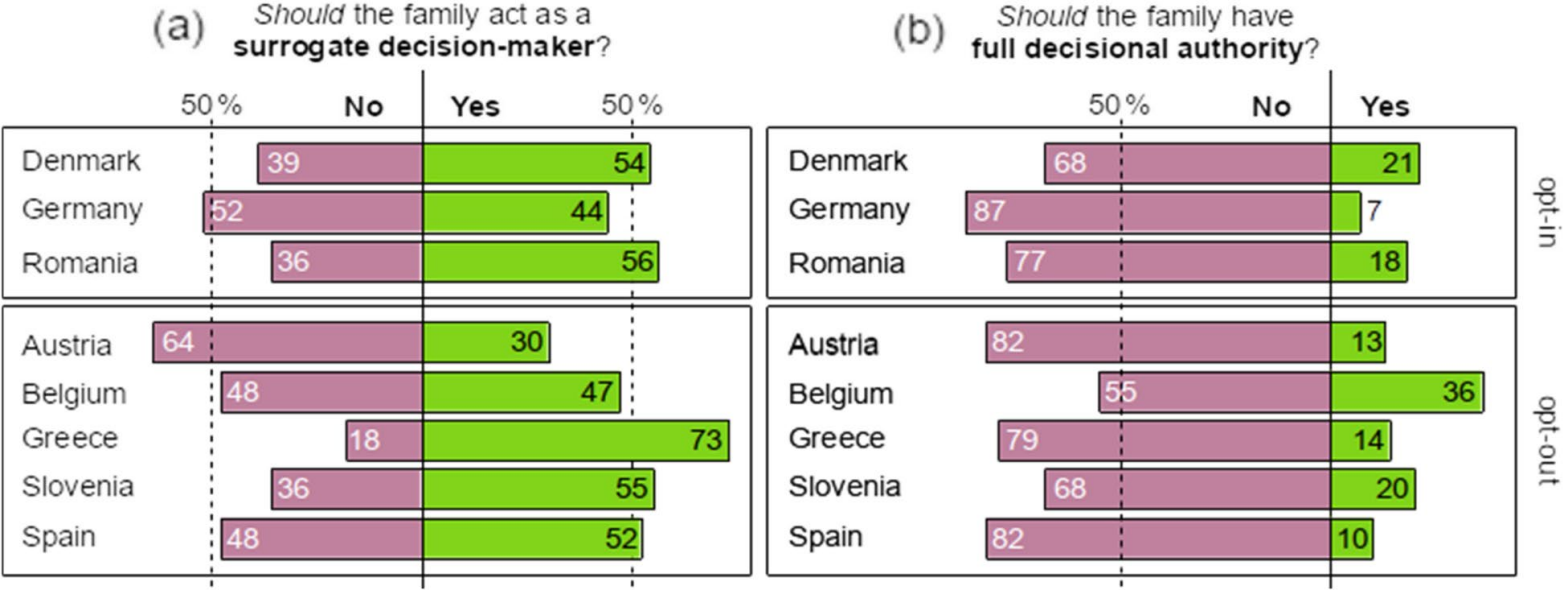
Respondents’ attitudes towards the role of the family. Percentages do not add up to 100 due to the omission of ‘Don’t know’ responses

**Fig. 3:**
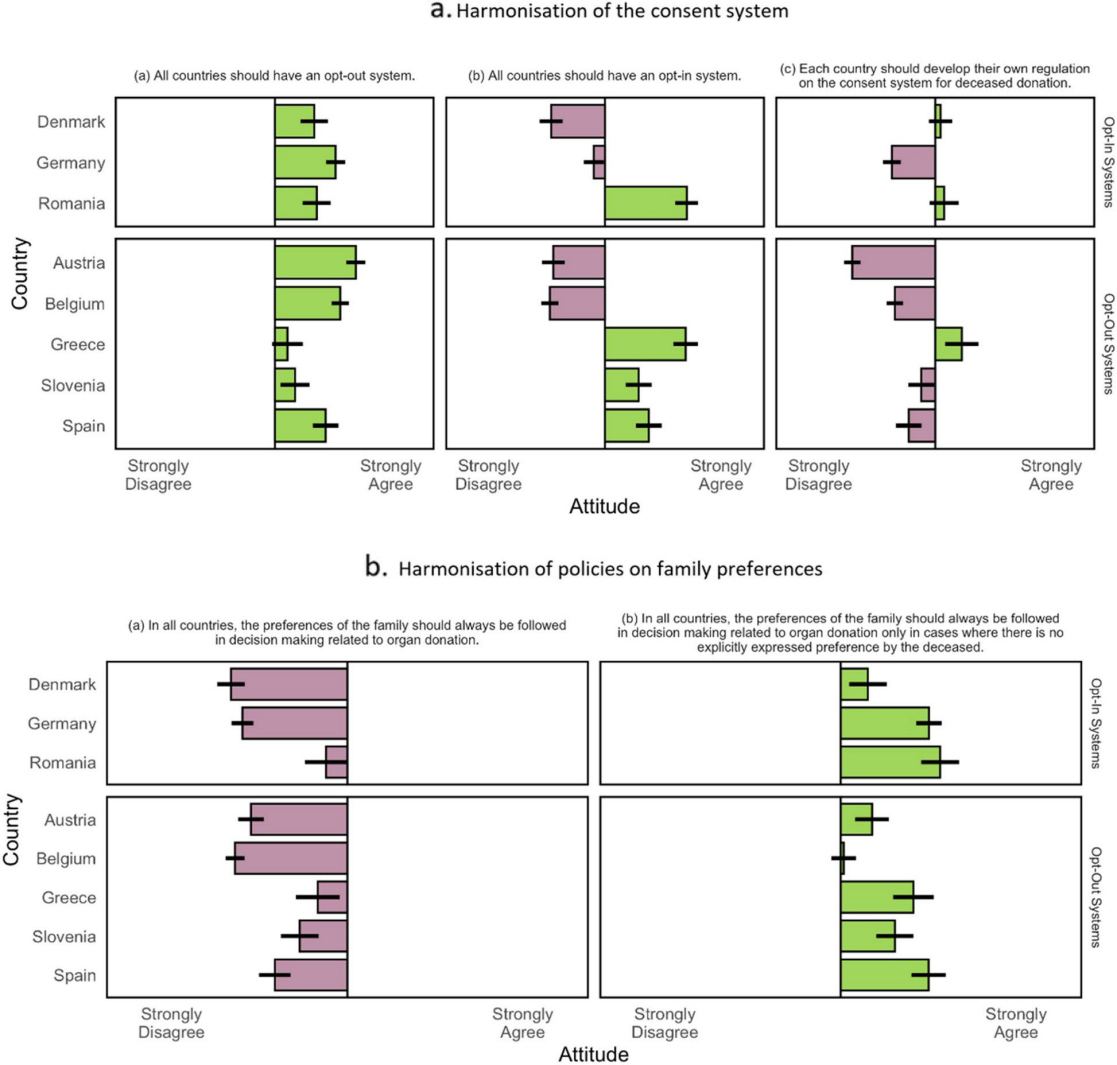
Views on policy harmonisation by country. A vertical line represents the scale midpoint, therefore leftward bars indicate mean disagreement and rightward bars indicate mean agreement. Whiskers on each bar display the 95% confidence interval. **a** Harmonisation of the consent system. **b** Harmonisation of policies on family preferences

We used a questionnaire that we developed and described in detail previously [15]. Briefly, the instrument includes questions about 36 items that focus on: (a) prior experiences, (b) knowledge and (c) personal opinions about regulation of organ donation, (d) which organs respondents would agree to donate and (e) accept to receive, and (f) their opinions on some issues raised in public discourse on deceased organ donation. An additional set of socio-demographic questions were included for contextualisation. Structure and questions were designed in accordance with previous survey studies in the field of organ donation [21]. The questions relevant for this paper (items 6, 7, 24, 25, and 26 of the questionnaire) are outlined in the Results section (see Figs. 1, 2, 3 and Tables 2, 3, 4). Further details—including the study protocol, the survey questionnaire, the participant information sheet, and the ethical review—are available as a Supporting Information File on the Open Science Framework repository [22]. This article follows the *Consensus-Based Checklist for Reporting of Survey Studies* (CROSS) [23].

The survey was conducted between October 2018 and November 2019 using convenience sampling methods (mailing lists, flyers) among university students in eight European countries: Austria, Belgium, Denmark, Germany, Greece, Romania, Slovenia, and Spain. These countries represent a variety of cultural backgrounds, have in place different legal systems regarding deceased organ donation, including opt-in and opt-out models of consent, and grant the family different roles in the decision-making process (see Table 1). Students were recruited from the second year onward, with approximately half of the sample being health science students and the other half humanities and social science students. Students of health sciences are a frequently chosen group to identify morally relevant attitudes to issues in medical ethics, facilitating comparisons with the results of other studies. Details regarding the recruitment methods, sample composition, study design, and analysis approach are outlined elsewhere [15].

**Table 1:** Classification of the consent system and the role of the family as specified by the law in the eight countries surveyed by this study. Source: [24]

Country	Consent system	Role of the family
Denmark	Opt-in	L2: Surrogate
Germany	Opt-in	L2: Surrogate
Romania	Opt-in	L2: Surrogate
Austria	Opt-out	L0: No role
Belgium	Opt-out	L0: No role
Greece	Opt-out^a^	L2: Surrogate
Slovenia	Opt-out^b^	L2: Surrogate
Spain	Opt-out	L2: Witness

^a^ Although Greece is usually classified as opt-out, Morla-González et al. [24] consider it an opt-in system because organs cannot legally be removed without either the deceased’s consent or the family’s authorisation. However, to avoid confusion we will here follow the usual classification of Greece as opt-out

^b^ Slovenia cannot be clearly described as either opt-in or opt-out according to the definitions used by Morla-González et al. [24]. However, to avoid confusion we will here follow the usual classification of Slovenia as opt-out

### Statistical analysis

Descriptive and inferential statistical analyses were conducted in R version 4.1.2, using the *lme4* and *emmeans* packages. We used one-sample proportion tests against the null probability of 50% and one sample *t*-tests against the scale midpoint to infer whether a particular response constituted the dominant (/non-dominant) view.

To evaluate whether participants’ views about what the role of the family should be differed from their beliefs about what it in fact is, we conducted tests of the equality of paired proportions. Finally, we conducted two-sample *t*-tests to examine the relationship between participants’ attitudes toward the role of the family and their preferences regarding harmonisation.

Given our large sample size, power analyses indicated that univariate and bivariate analyses were very highly powered (> 99%) to detect small effects (i.e., Cohen’s *d* = 0.20), setting the alpha level to .05. Study data are available on the *Open Science Framework* repository [25].

### Re-categorising results regarding the role of the family

We probed participants’ awareness of and attitudes towards the role of the family in two different scenarios: (i) when the deceased had expressed no preferences and (ii) when the deceased had expressly consented to donate (either verbally or in written form, which includes registries, organ donor cards, living wills, etc.). In the first case, the wishes of the deceased are unknown to the medical team and the family may or may not be allowed to make a decision about whether or not to remove the deceased’s organs. In the second case, while the wishes of the deceased are or can be known to the medical team, the family may or may not be allowed to overrule the deceased’s wishes. We re-categorised the resulting data based on Delgado and colleagues’ [18] four-level classification, according to which the family may either:

- (L0) Have *no role* at all in the decision-making process.
- (L1) Act only as a *witness* of the deceased’s preferences. Relatives can only inform or update the medical team about the last wishes verbally expressed by the deceased.
- (L2) Act as a *surrogate* decision-maker: Relatives can decide when the deceased did not.
- (L3) Act as the final decision-maker (*full authority*): Relatives can overrule the deceased’s expressed consent by blocking the removal of organs.

We assessed respondents’ knowledge of and attitudes towards the role of the family in their respective countries against a recent legal review of the countries surveyed [24].

## Results

A sample of 2193 university students, most of them aged 20 to 24, were included in the survey: Austria (339), Belgium (439), Denmark (230), Germany (424), Greece (159), Romania (190), Slovenia (190), and Spain (222). A majority (76%) were women, which partially reflects an over-representation of women in the disciplines concerned [26]. About half of the participants were enrolled in health-related study fields (for more details, see Rodriguez-Arias et al. [15] and the Supporting Information File [22]).

### Family involvement in organ removal decision-making

#### Awareness of the role of the family

A significant majority of students (ranging from 56% in Austria to 95% in Denmark) reported that, when the deceased had not expressed any preference, the family can act as a surrogate decision-maker (L2) (Fig. 1a) according to a one-sample proportion test against 50%, *p* < .001. This answer is incorrect according to the law in place in Austria, Belgium, and Spain. Austria is the only country where a considerable minority of students (23%) reported that it is not the family but the medical team who eventually decide in this situation. By contrast, when the deceased had expressed their decision in life, participants revealed greater disagreement (Fig. 1b). Overall, in a one-sample proportion test, significantly fewer than 50% reported that the family has full decisional authority (L3), *p* < .001.

#### Attitudes towards the role of the family

We asked respondents what they believe *should be* the role of the family in their country. Overall, in a proportion test against 50%, participants were ambivalent about the role families should play when the deceased had failed to express any preference, *p* = .13, notwithstanding variations between countries (Fig. 2a). In Denmark, Greece, Romania, Slovenia, and Spain, most participants supported the family’s capacity to make a decision when the deceased had not; in Austria and Germany, most participants opposed it; and Belgian participants were divided on this issue. With regard to the family’s full authority (L3), a simple majority in Belgium (55%) and a large majority in the rest of countries (ranging from 68 to 87%) stated that the family should *not* be allowed to overrule the deceased’s decision (Fig. 2b). When the deceased had expressed a preference in life, most respondents consider that the family should either not be consulted (L0) at all or just be allowed to witness/update the deceased’s last wishes (L1), *p* < .001.

#### Re-categorising awareness of and attitudes towards the role of the family

As summarised in Table 2, re-categorising our data according to the four incremental levels of family involvement [18] showed that most Danish and German respondents correctly believed, and most Austrian, Belgian, and Spanish respondents incorrectly believed that the family can (only) act as a surrogate decision-maker (L2). Romanian and Slovenian respondents hesitated between two roles: surrogate (L2), which is the correct answer, and full authority (L3). Most Greek respondents considered that the family has full authority (L3), which is incorrect.

**Table 2:** Re-categorising survey answers about knowledge of and attitudes towards the role of the family according to Delgado et al. [18]. The “national regulation” columns refer to the legal model of individual consent and the legal role of the family in each country as reported in Table 1

	National regulation on		Participants’ knowledge of and attitudes towards the family’s role	
Country	Consent	Family’s role	Beliefs (***knowledge***)	Preferences (***attitudes***)
Denmark	Opt-in	L2: Surrogate	L2: Surrogate	L2: Surrogate
Germany	Opt-in	L2: Surrogate	L2: Surrogate	L1/L2: Witness/Surrogate
Romania	Opt-in	L2: Surrogate	L2/L3: Surrogate/Full authority	L2: Surrogate
Austria	Opt-out	L0: No role	L2: Surrogate	L1/L2: Witness/Surrogate
Belgium	Opt-out	L0: No role	L2: Surrogate	L2: Surrogate
Greece	Opt-out	L2: Surrogate	L3: Full authority	L2: Surrogate
Slovenia	Opt-out	L2: Surrogate	L2/L3: Surrogate/Full authority	L2: Surrogate
Spain	Opt-out	L2: Witness	L2: Surrogate	L2: Surrogate

Most respondents in Belgium, Denmark, Greece, Romania, Slovenia, and Spain considered that the family should act as a surrogate decision-maker (L2), while in Austria and Germany they hesitated between two roles: witness (L1) and surrogate (L2).

We then compared respondents’ beliefs about what *is* the case in their country with what in their opinion *ought* to be the case for each level of family involvement. On the one hand, a vast majority (92.4%) of those who answered both questions thought that the family *can* act as a surrogate decision-maker (L2). This group of respondents, however, was ambivalent about whether the family *should* act as such (Table 3). On the other hand, a majority (64.8%) thought that the family *cannot* override the deceased’s wishes and a larger majority (81.9%) considered that the family *should not* have such authority (Table 4). In both cases, the proportion of participants who believed the family should exercise a given role was lower than the proportion who believed the family did in fact play that decision-making role, according to paired-samples equality of proportions tests, both *ps* < .001.

**Table 3:** Comparison of knowledge of and attitudes regarding whether or not the family can and should act as surrogate decision-maker. Numbers of respondents and percentages of the total of respondents who answered both questions are shown

L1. Surrogate decision-making		Should the family act as a surrogate decision-maker?		
		No	Yes	Total
Can the family act as a surrogate decision-maker?	No	109 (6.2%)	25 (1.4%)	134 (7.6%)
	Yes	735 (41.6%)	897 (50.8%)	1632 (92.4%)
	Total	844 (47.8%)	922 (52.2%)	1766 (100%)

**Table 4:** Comparison of knowledge of and attitudes on whether or not the family can overrule a deceased’s decision to donate organs. Numbers of respondents and percentages of the total of respondents who answered both questions are shown

L2. Full decisional authority		Should the family have full decisional authority?		
		No	Yes	Total
Can the family have full authority?	No	921 (57.2%)	123 (7.6%)	1044 (64.8%)
	Yes	397 (24.7%)	169 (10.5%)	566 (35.2%)
	Total	1318 (81.9%)	292 (18.1%)	1610 (100%)

Overall, it seems respondents consider that the family has a greater role than it should have in both cases. However, when comparing those who were satisfied with the family’s current involvement—because their beliefs and preferences coincide—with those who were unsatisfied, results show a slightly different pattern. A majority of respondents were *satisfied* with the family’s legal role in general and, in particular, with both its capacity to act as a surrogate decision-maker (Table 3, YES/YES: 50.8%) and its incapacity to overrule the deceased’s expressed wishes (Table 4, NO/NO: 57.2%). Among respondents who were *unsatisfied* with the family’s legal role, there is an asymmetry between those who would like less family involvement than what they believed is the case (YES/NO: 41.6 and 24.7%) and those who, on the contrary, would like more family involvement (NO/YES: 1.4 and 7.6%).

### Views on policy harmonisation of the consent regime

We enquired about participants’ views on harmonising consent policies and family involvement in organ donation decision-making within Europe. Participants were presented with five statements about the consent system (see Fig. 3a) and the role of the family (see Fig. 3b). For each statement, participants indicated their agreement or disagreement on a six-point scale ranging from 1: ‘Strongly Disagree’ to 6: ‘Strongly Agree’. A series of one-sample *t*-tests against the scale midpoint revealed the following aggregate tendencies: participants were (i) significantly in favour (grand mean = 4.41) of a harmonisation of opt-out systems, *t* = 25.12, *p* < .001, (ii) divided regarding the harmonisation of opt-in systems (grand mean = 3.39), albeit slightly opposed, *t* = − 2.83, *p* = .005, and (iii) clearly opposed (grand mean = 2.98) to the idea of national sovereignty over consent systems, *t* = − 14.49, *p* < .001.

A closer look at the national means revealed a more intricate pattern: As shown in Fig. 3a, participants from Austria, Belgium, Denmark, and, to a lesser degree, from Germany disapproved of establishing an opt-in system, while favouring an opt-out system as the preferred option for all countries. In Greece, Romania, Spain, and Slovenia, we observed support for the harmonisation of the opt-in consent system. This view, however, did not preclude approval of the opt-out system, which was also viewed favourably in these countries. Students in Austria, Belgium, and Germany expressed a stronger disapproval of the idea that each country should develop their own regulation, while a significant minority in Greece expressed a preference for national discretion.

With regard to the role of family preferences in deceased organ donation decision-making (Fig. 3b), in the aggregate, participants tended to oppose (grand mean = 2.53) a harmonisation of full authority, *t* = 32.31, *p* < .001, but supported (grand mean = 4.10) a harmonisation of the family’s surrogate decision-making role, *t* = 17.42, *p* < .001. In all countries except Belgium, there was substantial support for the view that family preferences should always be followed when—and only when—the deceased’s preferences are unknown. Conversely, there was substantial opposition to the view that family preferences should always be followed regardless of the situation.

Finally, we ascertained that participants’ views about harmonisation were in accordance with their personal preferences. Participants who thought the family *should* be granted full authority were also more likely to support the harmonisation of this policy throughout Europe (*M*_Yes_ = 3.00, *M*_No_ = 2.40; two-sample *t* = 7.73, *p* < .001)—although both groups were opposed to harmonisation on average. Similarly, participants who believed the family should exercise a surrogate decision-making role were more likely to support the harmonisation of this family role throughout Europe (*M*_Yes_ = 4.45, *M*_No_ = 3.78; two-sample *t* = 9.62, *p* < .001)—although both groups supported harmonisation in this second case.

## Discussion

### Statement of principal findings

Respondents from opt-in countries may have a better awareness of the legal role of the family than those from opt-out countries. Although a majority of respondents were satisfied with the family’s legal role, those unsatisfied preferred to limit family involvement. There is overall division on whether families should have a surrogate role, and substantial opposition to granting them full authority. Overall, respondents were opposed to the idea of national sovereignty over consent policies. They favoured opt-out policy harmonisation and were divided over opt-in. Their views on harmonisation of family involvement were consistent with their personal preferences.

### The role of the family and its impact on decision-making regarding organ donation

With regard to knowledge of national laws, most participants’ responses in opt-in countries were correct (i.e., in Denmark and Germany) or hesitant (Romania), while most participants’ answers in opt-out countries were incorrect (i.e, in Austria, Belgium, Greece, and Spain) or hesitant (Slovenia). In Romania, the law itself might not be very clear about this issue, and categorising Slovenia as opt-out rather than opt-in seems to be problematic as well (see [24]), which may partially explain why participants hesitated between two family roles.

These results are consistent with studies suggesting that people in opt-in countries have better awareness of the consent policy for organ donation [16]. Our survey data regarding participants’ knowledge of and attitudes towards the consent regime in place in their own country (opt-in or opt-out) were recently presented to support a theoretical tool for assessing governance of national policies on organ procurement [15]. Briefly, these data indicated that a majority of participants knew which consent system is in effect in the country where they live. However, in Spain, Greece, and especially Slovenia, most participants were unaware about their opt-out legislation (for detailed results, including hitherto unpublished survey data from Romania, see Supporting Information File, Figs. 1 and 2 [22]).

With regard to attitudes, most respondents gave precedence to the individual’s decision over family preferences in case of a conflict between the wishes of the deceased and the preference of their relatives. However, they tended to accept the intervention of the family when this does not undermine the individual’s autonomy. These results are consistent with previous studies showing public support for the role of relatives as surrogate decision-makers [17, 27]. However, studies on this topic often engender conflicting results. For example, two recent studies on medical students’ attitudes conducted at the same Polish university 2 years apart revealed support for family involvement (71% agreement) in 2018 [28], and opposition (26% agreement) only 2 years later in 2020 [29]. The existence of conflicting evidence calls for more representative and comparative longitudinal studies to provide a clearer picture of attitudes towards these questions, and their fluctuation over time.

Our study also suggests that, among those who are dissatisfied with the role they believe – correctly or incorrectly – relatives have in their respective countries, a majority would prefer to reduce family involvement. The age and assumed family status of our sample (i.e., primarily young university students) might influence their view on individual autonomy and the role of the family in organ donation. To document this, we encourage further research into whether parenthood, or aging processes more generally, affect individual attitudes on this issue.

Several countries have recently implemented laws or legal amendments to reduce the next-of-kin’s decisional authority. In 2006, the United States (opt-in) amended its law, the Uniform Anatomical Gift Act, to prevent relatives from overruling the deceased’s first-person authorisation. In 2007, Belgium (opt-out) modified its Law on Organ Removal and Transplantation by removing the option of family opposition. Other opt-out countries, such as Uruguay (Law 18.968, 2013), Colombia (Law 1805, 2016), France (Law 2016–41, 2017), and Argentina (Law 27.447, 2018) went a step further by legally preventing relatives from making any decision at all, even when the deceased failed to express any preference. Such legal changes are ethically delicate because of the trade-offs to be made between respect for the autonomy and the posthumous interests of the deceased, the autonomy and the interests of the deceased’s survivors, and the pressing interests of patients on the waiting list and of society as a whole [30].

There is a need for further investigation to better understand the impact of family (non-)intervention on organ donation rates, its impact on public trust in the organ donation and transplantation system, and its moral as well as social acceptability [9]. Similarly, more research and public discussion are required of whether donor families potentially hinder organ donation [31] or whether, on the contrary, they view donation as meaningful and thus facilitate donation, as Danish research suggests [32].

### Views on policy harmonisation

The European Commission promoted the harmonisation of national guidelines and policies as part of its Action Plan (2009–2015) [4]. Several actions have been undertaken to increase standardisation, including common management of waiting lists, registries for living donors, and specific training and twinning activities, which involve actions provided by EU projects or joint actions for transferring operational and educational expertise between member states. However, no attempts regarding harmonisation of consent models for deceased organ removal have been made so far. This may be because EU member states are at different stages in developing donation and transplantation programmes. Moreover, differences in culture and religion are known to affect attitudes towards organ donation [12] and may also affect acceptance of a harmonised model for consent and family involvement.

Assessing public attitudes and preferences regarding consent for organ removal and the role of families in the donation process may be necessary when considering the feasibility and desirability of a common European policy. To our knowledge, this is the first study to explore this topic. Our results suggest, first, that there is no strong opposition among participants against a common policy of consent for deceased organ donation, with the exception of Greece, where a slight preference for individual national policies was expressed. Second, the preferred common policy is presumed consent, which received approval from a majority of participants in all countries involved. Third, regarding harmonisation of the role of families, this study confirms previous evidence from Europe reflecting an overall preference for the surrogate role over the full authority of relatives [17]. Outside Europe, a majority of Australian students think that a soft opt-out policy (i.e., where relatives act as surrogate decision-makers) is the most effective policy [27].

An increasing harmonisation of consent policies is already underway, with a growing majority of European and non-European countries adopting an opt-out model. The results of our present study suggest that this trend enjoys substantial public support. Regarding the role of the family, there appears to be more variation in national legislation than in clinical practice [18]. In most countries, the family can act as de facto surrogate decision-maker and, in many countries, as final decision-maker (i.e., full decisional authority). Most students seem to be comfortable with this. A harmonisation of laws that would be consistent with current practices might therefore garner their support.

## Limitations

A systematic review has shown that most general public surveys on the role of the family in decision-making on deceased organ donation have been conducted in opt-in countries, usually by governments or national transplant organisations, while most surveys on students have been conducted in opt-out countries [16, 17]. Therefore, it is uncertain whether university students’ views on organ donation in a given country vary from those of the general population. More particularly, assessing people’s knowledge of the role of the family is complicated because of the difficulty of determining what constitutes the correct answer. Laws are often vague, ambiguous, tacit or silent about what relatives can and cannot do in different circumstances [24]. Moreover, in most opt-in and opt-out countries, relatives are usually granted a more important role in clinical practice than in law [18, 33]. In addition, actual practices may vary from place to place within the same country [34].

Because this study is limited to university students and a majority of participants are women, results cannot be generalised to the whole population. Gender bias can be partially explained by high female–to–male ratios in the sampling frame, especially among majors in health sciences (54–72%; vs. 39–62% for humanities/social sciences), and high female to male ratios among university students in Europe (72% for health sciences students; 64% for humanities/arts/social sciences students) [26]. In addition, there may be sex differences in non-response bias, including higher retention and completion rates among women than men [35]. The questionnaire length may also have selected for highly motivated participants.

## Conclusions

A growing number of countries around the world are moving from opt-in to opt-out policies. Our study suggests that if European countries were to harmonise their consent policies, an opt-out system that gives families a surrogate decision-making role, which—either de jure or de facto—is already the case in many countries, may have significant public support.

However, our results also reveal a weaker understanding of the family’s legal role among participants from opt-out countries compared to those from opt-in countries. The observation that, despite their higher education, university students still have difficulty understanding how consent and decision-making work in an opt-out system underscores the need for better information dissemination among the general public. This needs to be considered when transitioning towards opt-out policies in order to increase donation rates, and is relevant for clinicians, who play a central role in the decision-making process and are tasked with balancing the interests of potential donors, families, and patients on the waiting list.

To our knowledge, this study provides for the first time a suitable survey tool for international cross-sectional assessment of knowledge and attitudes towards the role of the family in decision-making on deceased organ donation and harmonization of consent policies. As more data on the general population would provide better evidence, we recommend further support for time- and budget-intensive cross-European studies.

## Supplementary Information

**Additional file 1.**

## Data Availability

The datasets generated and analysed during the current study are available on the *Open Science Framework* repository: https://osf.io/x98mp/?view_only=5388c94b5c9c44d08659a2b6a97dadeb [Accessed 11 July 2022].

## Abbreviations

ELPAT-ESOT: Ethical, Legal, and Psychosocial Aspects of Transplantation – European Society for Organ Transplantation
EU: European Union
PMP: Per million population
