# Supplementary material for: Attitudes of European students towards family decision-making and the harmonisation of consent systems in deceased organ donation: a cross-national survey
Source: BMC Public Health. 2022 Nov 15;22:2080. doi: 10.1186/s12889-022-14476-z (PMC9664433; doi:10.1186/s12889-022-14476-z)
Supplement: Supplementary file 1 — Additional file 1. [file 12889_2022_14476_MOESM1_ESM.pdf]

Supporting information file

Attitudes of European students towards family decision-making and the harmonisation of consent to deceased organ donation: A comparative cross-sectional study

Contents

Study-protocol 2
Survey questionnaire 14
Participant Information Sheet 24
Recruitment process 25
Sample composition 26
Additional results on consent policies for deceased organ donation 28

Study-protocol

International Student Survey on the Knowledge and attitudes towards donation and transplantation of organs/body parts (ISSATO)

Principal Investigator:
Prof. Dr. Silke Schicktanz
University Medical Center Göttingen, Department of Medical Ethics and History of Medicine
Humboldtallee 36, 37073 Göttingen, Germany
Silke.schicktanz@medizin.uni-goettingen.de

Study coordinator:
Dr. Sabine Wöhlke
University Medical Center Göttingen, Department of Medical Ethics and History of Medicine
Humboldtallee 36, 37073 Göttingen, Germany
Sabine.woehlke@medizin.uni-goettingen.de

Cooperation partners (in alphabetic country order):

Belgium
Kristof Van Assche
University of Antwerp, Research Group Personal Rights and Property Rights, Faculty of Law, University of Antwerp, ANTWERP – Belgium

Denmark
Anja Marie Bornø Jensen
University of Copenhagen, Centre for Medical Science and Technology Studies, Dept. of Public Health, COPENHAGEN – Denmark

Greece
Prof. Thalia Bellali RN-Dipl.Psych
"Alexander" Technological Educational Institute, Dept. of Nursing, THESSALONIKI - Greece

Spain
David Rodríguez-Arias
University of Granada, Dept. of Philosophy, GRANADA - Spain

Alberto Molina Pérez
University of Granada, Dept. of Philosophy, GRANADA - Spain
Spanish National Research Council, Institute for Advanced Social Studies (IESA-CSIC), CORDOBA - Spain

Janet Delgado Rodriguez
University of Granada, Dept. of Philosophy, GRANADA - Spain

Romania
Andrei C. Holman
Alexandru Ioan Cuza University, Faculty of Psychology and Educational Sciences, IAȘI – Romania

Mihaela Frunza
Babes-Bolyai University, Department of Philosophy, CLUJ – Romania

Slovenia
Tanja Kamin
University of Ljubljana, Faculty of social sciences, LJUBLJANA – Slovenia

1. Summary:

Several European countries have recently changed or considered changing their organ procurement policies and also the model of consent for organ donation. The aim of this international quantitative survey is to investigate for the first time knowledge and attitudes of lay persons in a comparative and in an in-depth manner. The selection of countries (BE, DK, GER, GRE, ES, SI, ROM) does also represent different systems of consent for post-mortem organ donation in each country.

Students are selected as first target group because they represent an educated section of society, and socio-political attitudes are formed in their age. Furthermore, access to students is easier than to other social groups and make such as study more feasible. We will include students from health sciences (nursing/medical/public health) and from non-health sciences (social sciences, anthropology, philosophy, social work, sociology, political science).

Our study wants to test for social factors such as national background, study topic, gender, and personal affectedness. Based on the results, the questionnaire will further be developed in terms of its suitability for a representative European population survey.

2. Study Goals

The aim of the planned survey is to analyse the knowledge and the attitudes of students across different European countries about the regulation of post-mortem organ donation. A special focus is put on ethical and social questions regarding the motivation to donate/not donate of different organs/body parts, the moral assessment of consent procedures including the role of the family, and attitudes towards information policies and satisfaction/trust with the current transplantation system. Social factors to be tested are: national background, study topic & year of study, gender, religion, and personal affectedness.

Furthermore, we want to test how general attitudes to donate/not donate correlate with attitudes towards different consent systems. The goal of this quantitative survey is to shed light on key ethical and social challenges that organ transplantation raises with a focus on laypersons' attitudes in different European countries. This study will strengthen the public discourse and give ethical recommendations. The study will also serve to validate the questionnaire to test for population representative surveys.

3. Scientific Background

Organ transplantation has been described as "a perpetual competition between the people who need organs and the rest of us who have them" (Radcliffe Richards 2012). Arguably, deceased organ donation prototypically differs from other biomedical practices in that those who can help others through a personal sacrifice have been declared *dead*. Some bioethicists have argued that death destroys all individual interests, thus making organ donation no sacrifice at all, and *pre-mortem* information and consent morally superfluous (Hershenow & Delaney 2009). International policies have expressed a clear support for organ transplantation by implementing different initiatives to address the shortage of organs (European Parliament 2006). However, none has fully endorsed organ conscription — the automatic procurement regardless of consent—, suggesting that all countries assume, although in different agrees, that potential organ donors —or their family members— have at least *some* claims on their bodies after death (Childress & Liverman 2006).

Understanding individuals' level of knowledge and their attitudes towards the legal requirements to be organ donors therefore becomes important. Surveys are an important initial step to gauge relevant factors for the willingness or unwillingness to donate an organ under specific conditions. While they are seldom exact predictors of behaviour, surveys can still provide very important insights into public common sense (Mossialos et al. 2008; Rithalia et al. 2009) and reveal public moralities towards organ donation as well as towards the incentive debate (Hoeyer et al. 2013; Schicktanz et al. 2013; Inthorn et al. 2014).

In Europe, a common strategy to address organ shortage is through modification of the legal model of consent for *post-mortem* organ procurement to an opt-out system (i.e., presumed consent). Hence, individuals are automatically considered as donors after death unless they have explicitly opted out during their lifetime.

Some European countries have been using opt-out for a long time (e.g., Spain, Austria, Belgium). Other regions have recently considered or achieved legislative changes (e.g., Greece, Romania, Iceland, Wales, The Netherlands, and France), whilst others have recently carried out (Scotland (Christe 2017)), or are currently carrying out (England (Iacubucci 2017)) public consultations regarding a change to the opt-out system.

However, considerable variety within Europe legislation still exists. This can be explained by several factors: different historical and normative backgrounds, policy makers' uncertainty as to which model would be more effective in a given culture, and concerns that nudging people into donation might be perceived as disrespectful and thus be counterproductive. In 2008, a proposal for a Directive of the European Parliament and of the Council of Europe stated that "consent for procurement is as a general rule regulated by Member States in very different ways", and that "this is a very sensitive field" which "raises a number of ethical concerns that falls within the competence of the Member States" (European Parliament 2006).

Additional strategies include creating organ donation (OD) registries, mandatory choice policies (by which people are required by law to state in advance whether or not they are willing to donate), or removing families' capacity to veto OD. In opt-in countries, where a donor card or registration and information and public knowledge is essential, people will only donate if they are aware of their active part in consenting. In opt-out countries, some ethicists, too, consider broad publicity and information as a moral prerequisite of such policies, given that autonomous choices require understanding the consequences of expressed and non-expressed preferences (Rodriguez-Riaz, Morgan 2016; Nordfalk et al. 2016).

Studies that are more recent suggest that people's awareness of the model of consent for OD in their country increases their willingness to donate (European Commission 2013, Shepherd & O'Carroll 2013). Many studies focused on citizens' attitudes towards organ donation (e.g., willingness to donate) (Bastami et al 2013; de Groot et al. 2012; Haugland et al. 2016; Irving et al. 2012; Simpkin et al. 2009; Shah et al. 2015) and the impact of different consent systems on donation rates (Gimbel et al. 2003; Healy 2005; Abadie & Gay 2006; Neto et al. 2007; Coppen et al. 2008; Coppen et al. 2010; Mossialos et al. 2008; Rithalia et al. 2009; Bilgel 2012; Shepherd et al. 2014; Hawkes 2017). However, the level of public knowledge and support of national policies on consent for deceased organ donation has not been analysed on an international comparison stage.

A German study focused on attitudes from medical and economist students showed that a different image of the body has an influence on attitudes towards organ donation. A large majority of students (84.1 %) favoured a holistic concept of the body in which the human body signifies more than just the sum of its parts. Despite this rather clear result, there were significant gender differences: More women (87.6 %) than men (80.6 %) have a holistic conception of the body. While only 6.3 % of women agreed with this statement, 15.9 % of men agreed (sig. $p < 0.001$. Medical students were also more in favour of this body concept (8.3 % economics, 12.5 % medical, sig. $p < 0.001$), while economics students showed a tendency of being unsure about their answers (15.1 %). Economics students also showed this uncertainty with regard to the idea that the heart is the location of the soul and should not be transplanted, 26.0 % were unsure about this. Uncertainty was also relatively high regarding the question of whether certain body parts are believed to be significant for identity. Thus, 25.3 % of respondents were uncertain whether specific organs determine a person's individuality. 41.1 % of respondents disagreed with this statement and 33.7 % agreed (Wöhlke et al. 2016).

Moreover, the study also illustrates what type of organ respondents would accept in case of severe organ failure. There was a higher preference for organs of human origin (postmortem or living), than for animal or artificial organs. Women opted for a living organ significantly more often (76.8 %) than men (71.9 %) ($p = 0.014$). Respondents were more sceptical about other alternatives, e.g., animal organs, organs from stem cells, or a machine. Yet, more than half of the participants

stated that they would accept an organ grown from stem cells, but while 76.1 % of men would accept such an organ, only 58.9 % of women would agree to this option. Here, women were significantly more uncertain (32.3 %) than men (16.6 %) ($p < 0.001$). The same level of uncertainty emerged on the question of whether an artificial machine would constitute an adequate surrogate organ (31.1 %) or about the option of an animal organ (36.1 %). Apart from the origin of an organ, acceptance levels may also vary with respect to the type of human organ. The answer options ranged from fully acceptable to do not accept at all. The kidney showed the highest level of acceptance, scoring 1.3 (whole data set), as well as the liver with 1.4, followed by lung transplant and the heart (1.6). Respondents were uncertain about the option of accepting face transplantation (3.8) and the transplantation of genitals, which showed a mean of 4.3. Strikingly, the mean values of all options (except for the pancreas) showed that men were more willing to accept any kind of organ than women. While answers with regard to the kidney, liver, and lung only showed a slight tendency towards differing opinions, this difference became more pronounced in the option of accepting a heart transplant (1.5 for men, 1.8 women, sig. $p = 0.003$). When it comes to externally visible organs, differences between men and women were even more significant. Values on the acceptance of a cornea transplant ranged from 2.0 among men to 2.3 among women (sig. $p = 0.003$). These differences increased even more in responses to the option on the acceptance of receiving a whole eye transplant (2.8 men; 3.6 women, sig. $p < 0.001$), acceptance of single limbs (2.8 men; 3.5 women, sig. $p < 0.001$), a full face (3.4 men, 4.1 women, sig. $p < 0.001$), or a transplantation of genitals (3.9 men, 4.6 women, sig. $p = 0.001$). Overall, the acceptance of an organ was highest for human organs and those organs that are invisible within the body, with men showing greater acceptance of transplantation than women (Wöhlke et al. 2014).

Only one systematic review by Rithalia et al in 2008, followed by an update in 2012, exists on a comprehensive analysis level on attitudes towards presumed consent. The original review found 10 surveys on public views in European countries between 1950 and 2008, 8 of which from the UK. It is acknowledged that "important methodological detail was not available" for most of them and concluded that there was limited and inconclusive data to answer the question of whether the public supports the system of opt-out. The review update found 6 additional surveys, 5 of which from the UK and one from Ireland, and reached a similar conclusion. Rithalia et al.'s systematic review contributed to a UK Taskforce recommendation "that an opt-out system should not be introduced in the UK" because of its "potential to undermine the concept of donation as a gift, to erode trust in NHS professionals and the Government, and negatively impact on organ donation numbers" (Organ donation Taskforce:34). This review did not address other models of consent for OD apart from opt-out.

The ELPAT working group on "Public Issues" (Molina-Perez A., Rodriguez- Arias D., Delgado J., Morgan M., Frunza M., Randhawa G., Reiger J., Schicks E., Woehlke S., Schicktanz S.) conducted a systematic review to examine public knowledge and attitudes towards different models of consent for post-mortem organ donation in Europe. The manuscript: Public knowledge and attitudes towards models of consent for organ donation in Europe. A systematic review is actually under revision [Edit: it has now been published; doi: http://dx.doi.org/10.1016/j.trre.2018.09.001]. It reports that awareness of the model of consent was higher in *opt-in countries* than in opt-out countries. Qualitative studies also suggest that among people who claim to be aware of the consent system, understanding of the consent system is often limited. Studies indicate that a majority of respondents agreed with the opt-in system, regardless of the consent model in place in their own country. The majority of respondents in opt-in countries also agreed with mandatory choice, where people are mandated to register a decision, either in favour or against organ donation. Results concerning the opt-out system are more variable. This review has revealed great differences within European countries regarding data availability on public knowledge and attitudes towards models of consent for OD and demonstrated considerable *between* country variation in attitudes and preferences. This review also suggests that, while most people support any specific consent system when asked about it separately, they tend to prefer opt-in and mandatory choice to opt-out when they are offered two or more options.

The large student survey conducted in 2008/09 (see above) to explore complex attitudes towards organ donation (Inthorn et al. 2014; Wöhlke et al. 2016) was repeated in 2014/15 to test for respective correlations (total n = 1,403) (Schicktanz et al. 2017). Apart from the educational bias, students are a very relevant population for two reasons. Firstly, young and educated adults are more positive towards organ donation than other groups (Federal Centre for Health Education 2015); therefore, it is

easier to motivate them to take part in complex and detailed surveys. Secondly, late adolescence seems to be an important phase for developing social and political attitudes, which also might be very relevant regarding attitudes to organ donation.

The aim is to develop a questionnaire that allows interviewing relevant social groups and to analyse the indirect factors (e.g., relevant values on body concepts, definition of death, cultural values). In order to prepare such a survey, it is necessary to check the developed questionnaire in advance for efficiency, content accuracy, comprehensibility, and readability. For this purpose, a survey is carried out on a representative scale with students of health-related disciplines and non-health-related disciplines on an international level.

4. Aims of the study

4.1. Primary scientific question (formulated as hypotheses):

Differential Hypotheses: Differences between Specific Groups:

National Background

1. H0: There is no difference in knowledge and attitude between the students of different national backgrounds.
2. H1: There are national differences between students.
3. H2: National difference regarding opt-in vs. opt-out will impact participants' knowledge: In opt-in countries knowledge is higher about their regulation than in opt-out.

Medical students vs. non-medical students

1. H0: There is no difference between the medical students and non-medical students.
2. H1 More medical students favour post-mortem organ donation than non-medical students (especially with the increase of study time).
3. H2: Medical students favour the opt-out system as a legal regulation, while non-medical students prefer the opt-in system (yes answers to different forms of regulations).

Men vs. women

1. H0: There is no difference between men and women.
2. H1: More women favour post-mortem organ donation then men.

Personal affliction vs. non-personal affliction

Index personal affliction (sum of yes answers to personal environment to organ donation/ chronic organ disease)

1. H0: There is no difference according to personal affliction.
1. H1: Affected study participants are more likely than the non-affected to be in favour of organ donation and opt-out system.

4.2. Secondary goals:

Testing for related attitudinal factors:

- Connection between body concepts and attitudes to organ donation.
- Correlation between the knowledge about organ donation and the attitudes towards the legal rules for becoming a donor.
- Connection between trust in the medical system and the willingness towards organ donation.

Explorative hypotheses:

Identification of social factors (national background and cultural attitudes (e.g., body concepts/religion) as possible impact for different attitudes (for/against organ donation).
Identification of possible structures for one-sided behaviour (only favour opt-in solution) but no willingness to donate organs (cluster analysis, which characteristics distinguish this group from others)

Identification of possible structures for strongly negative behaviour (contra organ donation) and critical behaviour towards the medical system → are there clusters/subgroups in this case?

Further detailed evaluation strategies will be developed during the analysis.

5. Study Design & Schedule

5.1. Preliminary research and preparatory work

Since 2011, the majority of the collaborators has been working on related issues in the context of ELPAT (Ethical, Legal and Psychological Aspects of Transplantation - www.esot.org/ELPAT/home) working group "Public Issues". The principal investigator, Prof. Silke Schicktanz, has also extensive experience in conducting a survey with students on the subject of organ donation. In the winter term of 2008/2009, her team conducted a comprehensive survey with students on organ donation in Germany. In the winter term 2014/15, that survey was repeated. The survey comprises 55 question complexes in a closed response format to assess the knowledge and interest in post-term and living organ transplantation.[1] All partners involved in this international survey have experience with the topic of organ donation.[2]

In addition, the working group has been working on a systematic review over the past two years. Given the ongoing need to have public discussions and to tackle the issue of organ transplantation in Europe, the group has conducted a systematic review (2008-2017) to gain evidence on the knowledge and attitudes of the public towards the legal systems of consent and *post-mortem* donation for European countries. Assessing current public knowledge and attitudes towards different models of consent for organ donation is required to inform public policies regarding systems of OD. The manuscript: Public knowledge and attitudes towards models of consent for organ donation in Europe. A systematic review with the main results is currently under revision [Edit: it has now been published; doi: http://dx.doi.org/10.1016/j.trre.2018.09.001].

The survey contains 36 questions (see questionnaire attached). The following aspects were taken into account:

a) Experiences concerning organ donation and transplantation
b) Knowledge about the legal regulation of organ donation
c) Personal opinion towards organ donation and how it might be regulated
d) Personal opinion about different body concepts
e) The image of the human body
f) Personal views on the public discourse and politics towards organ donation
g) Prior experience on organ donation (incl. presence of a donor card)
h) Willingness to donate (to whom and under what conditions)
i) Willingness to accept an organ (by whom and under what conditions)
j) Attitudes towards the legal situation
k) Differences between men and women
l) Differences between medical students and non-medical students
m) Personal questions (including age, religion)

Filling out the survey will take 20 minutes approximately. We used a six-level Likert scale for attitude questions, and yes/no/don't know for knowledge as well as for simple questions on decisions or willingness.

---

1. Wöhlke, S, Inthorn, J, Schicktanz, S. The Role of Body Concepts for Donation Willingness. Insights form a Survey with German Medical and Economics Students, 2015. In Jox, RJ, Assadi, G, Marckmann, G (Eds.): Organ Transplantation in Times of Donor Shortage. Challenges and Solutions, Springer, p. 27-49; Inthorn, J, Wöhlke, S, Schmidt, F, Schicktanz, S. Impact of gender and professional education on attitudes towards financial incentives for organ donation: results of a survey among 755 students of medicine and economics in Germany, 2014, BMC Medical Ethics, 15, 56, 10.1186/1472-6939-15-56; Schicktanz, S, Pfaller, L, Hansen, SL, Boos, M. Attitudes towards brain death and conceptions of the body in relation to willingness or reluctance to donate: results of a student survey before and after the German transplantation scandals and legal changes, Journal of Public Health, 1-8, 10.1007/s10389-017-0786-3.
2. Brief selection of literature: Randhawa G, Brocklehurst , Pateman R, Kinsella S, Parry V. 'Opting-in or opting-out?'- the views of the UK's faith leaders in relation to organ donation. Health Policy. 2010 Jun;96(1):36–44.; Nordfalk F, Olejaz M, Jensen MB, Skovgaard LL, Hoeyer K. From motivation to acceptability: a survey of public attitudes towards organ donation in Denmark. Transplant Res. 2016;5:5; Hansen SL, Eisner MI, Pfaller L, Schicktanz, S. "Are You In or are You Out?!" Moral appeals to the Public in Organ Donation Poster Campaigns: multimodal and Ethical analysis. *HealthCommunicaton,* 2017 :1–15; Rodrıguez- rias D, Morgan M. "Nudging" Deceased Donation Through an Opt-Out System: A Libertarian approach or Manipulation? *Am J Bioeth* 2016;**16**:25–8. doi:10.1080e15265161.2016.1222022; Rodríguez-Arias, D.; Wilkinson, D.; Youngner, S.: How Can You Be Transparent About Labeling the Living as Dead? 2017, AJoB 17(5):24-25, 0.1080/15265161.2017.1299243.

5.2. *Translation*

The final version will be in English and each party will be responsible for translating it into their national/regional language as well as for validation. This includes a back translation by a third experienced/professional person (not from the field). The translated questionnaire must be pre-tested for comprehensibility (qualitative testing with 2-3 students).

Additionally, in May 2018 we conducted a small pilot study to check the feasibility and functionality, once the questionnaire is available online (around 10 students in the UK).

5.3. *Online Survey*

We use common software (LimeSurvey©) which is open source and offers a manual for scholars and is very low cost.

6. Study population / selection of students

Students of medical and non-medical sciences compose a frequently chosen group to identify morally relevant attitudes to medical ethical issues. This is certainly due to the easy availability, but also to the high practical relevance (i.e., physicians face ethical conflicts when making concrete medical decisions). At the same time, the inclusion of medical students allows for a comparison of our results with the results of other studies (e.g., with regard to national or cultural differences).

The comparison of students of medical sciences and students of non-medical sciences will allow a comparison with the study of Wöhlke et al. 2016; Inthorn et al. 2014, Schicktanz et al. 2017 & Sahin et al. 2015, in which significant differences were found in the acceptance of organ transplantation among students from different countries. At the same time, students of non-medical sciences may be considered as laypersons. Their feedback on intelligibility is therefore very relevant for the conception of the general population survey. Of course, due to the level of education and often the social background, students cannot be considered as representatives of the average population.

We aim at recruiting in each country/university at least 100 students of medical sciences and 100 students of non-medical sciences.

6.1. *Recruitment*

Students should not be recruited during lectures. The Survey is online and information about the online survey will be given via flyers and mailing lists. The participation to the survey is voluntary. Advertisements embedded at the end of lectures are possible.

The students fill out the survey online outside their studies or during the teaching breaks. Before beginning the survey, all participants will be informed about the aim of the study. At the end of the study information, each student agrees to the participation by ticking a checkbox and only then will be redirected to the survey. Participation can be interrupted or cancelled at any time until the end of the survey. Uncompleted survey data will not be saved.

IRB: Each cooperation-partner is responsible to check the local rules regarding IRB/ethics approval in his/her country. Because the survey will not be conducted during lectures, an IRB will probably not be necessary. According to the local ethics committee at the University Medical Center Göttingen, no formal approval for this kind of research is needed.

The participation to the survey is voluntary and anonymous. To get a higher response rate, we work with a small incentive (e.g., a raffle of 4 amazon vouchers in each country). Each cooperation-partner is responsible him/herself for this incentive. Anonymisation by the participants is ensured by selecting a cross-reference on the last page of the online survey, which can only be used once by each IP address. After redirecting to another webpage, participants can enter their contact details to participate in the raffle.

6.2. *Inclusion criteria:*

1. Beyond 18 years
2. Second study year onwards
1. Local students (from one university)
2. Health sciences students (nursing/medical/public health)
3. Social sciences/Humanities students (Anthropology, Economics, Humanities, Philosophy, Social work, Sociology)
4. Study topics with a gender-balanced population of students, within a 40/60 ratio

6.3. *Exclusion criteria:*

1. Below 18 years
2. 1st year students
3. Non-students
4. Students from fields of natural sciences such as physics, biology, chemistry, mathematics, engineering, informatics or from legal studies
5. Study topics with extreme gender bias

6.4. *Incentive*

Among the participants, in each country four vouchers (total 100 €) will be raffled (e.g., for an online shop, such as books). Anonymisation is ensured by the participants being able to select a cross-reference on the last page of the online survey, which can only be used once by each IP address. After redirecting to another webpage, participants can enter their contact details to participate in the raffle.

7. Organisation of the survey / Data management

The online survey will be conducted at the beginning of the winter term 2018/2019 in all cooperation countries. To that aim, we will use the survey-tool Limesurvey (https://demo.limesurvey.org/index.php/admin/authentication/sa/login). LimeSurvey can create multilingual surveys. We choose English as "base language" for our survey and can add several "additional languages". The texts for other languages <u>must all</u> be entered separately, there is no automatic translation. Sabine Wöhlke will get a group administrator account and can manage this part of creating the respective survey versions. A jointly formulated letter is provided to all cooperation partners for this announcement. Each cooperation partner is responsible for translating this letter into their respective language. Potential participants can access the survey via a link in this information letter.

In order to participate to our survey, the participants must first have a look at the study information. By clicking the checkbox, they will agree to participate and afterwards will be forwarded to the actual questionnaire.

There will be three reminder e-mails which will be sent every 8 weeks followed by an invitation to our survey to guarantee a highly response rate.

By using cookies, the software recognises if the same person tries to fill in the questionnaire again. Therefore, the risk of multiple data entry by one person can be reduced.

7.1. *Data collection and Data analysis*

Quality assurance:

All data collected by Limesurvey are automatically stored by the server of the GWDG (Göttingen / Germany) (https://www.gwdg.de/), the local IT-Partner of the University of Göttingen. Sabine Wöhlke as group administrator has an account and manages the data collection.

Statistical analysis

The statistical analysis is carried out by the trained specialists, Ivar Rodriguez Hannikainen, Department of Law, Pontifical Catholic University Rio de Janeiro (Brazil). Since 2018 Mr. Rodriguez Hannikainen is a Postdoctoral Fellow at the Department of Law, Pontifical Catholic University Rio de Janeiro

(Brazil). He has extensive experience in conducting quantitative methods and has collaborated with prominent experimental philosophers/psychologists interested in ethics, such as Joshua Green and Edouard Machéry.

After collecting the data, we get the data in usable from for SPSS. We will make different statistical comparison of the mean value comparison with Chi-square test or T-tests between the mentioned groups.

Further analysis of possible correlations between different attitude patterns as well as exploratory connections through Anova/classification tests.

7.2. Time schedule

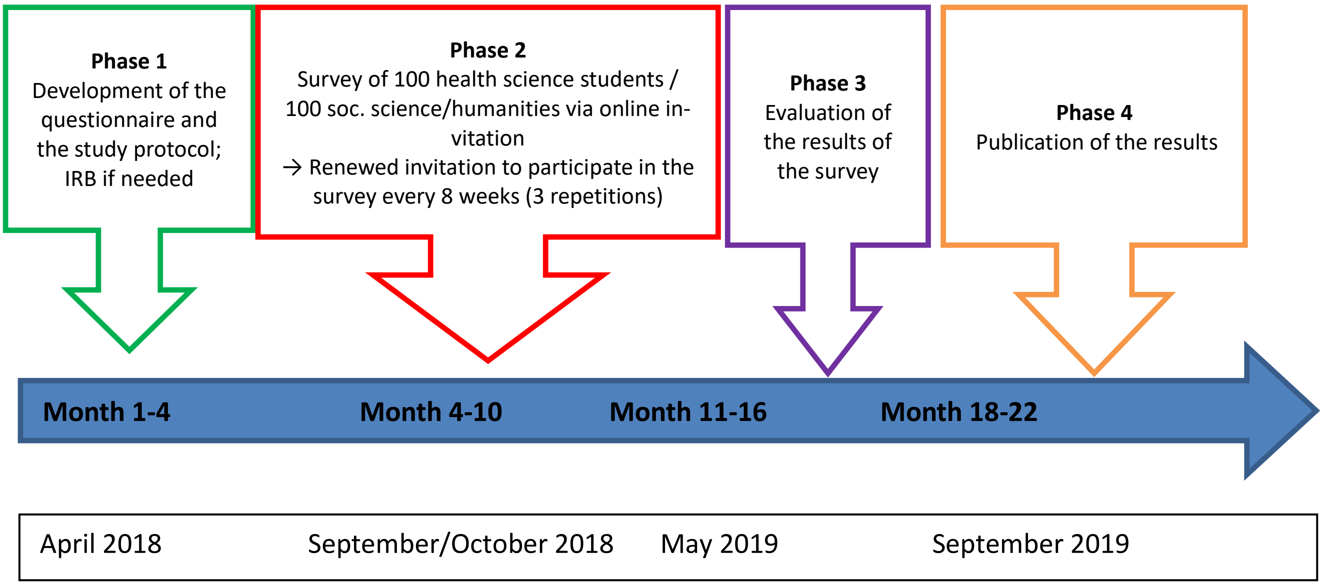

8. Ethical aspects & data protection:

Each cooperation partner is responsible for the clarification of an IRB (for his/her country) and will report this to the PI/study coordination.

All participating students will be informed about the aims, the data protection, and the publication of the anonymous results in written form. Thus, information will be based on this study protocol and also depend on national/local ethics standards.

After reading the study information, they will be asked to sign online by clicking a checkbox. By clicking, the participants will express their own, clear interest in participating in the study. This will be considered as their consent to the study.

Data collection of the survey and analysis is fully anonymised.

To increase the incentive to participate in the survey, four vouchers of 25 Euro will be raffled amongst those who fill in the questionnaire. Participation in the raffle is voluntary. Those who want to take part in the raffle will be asked to indicate their name and email address in an extra column. The collection of the latter will be fully disconnected from the survey data, i.e., no linkage between survey data and a person's name or email address can be established. Given that the survey approaches professionals/experts as participants, we regard the option of winning 25 Euro in a raffle as a rather small incentive that still complies with the ethical norm of voluntary participation in research. The personal data will be stored and processed under special protective measures (access, access and disclosure control, see e.g., German data protection law Annex § 9 BDSG).
The groups of students thus benefit from the conduct of the study by integrating their perspectives into the discourse, which is often dominated by experts, and by making it useful for the improvement and regulation of the practice.

8.1. *Cancellation of the study*

Participants have the opportunity to cancel their participation at any time *during* the survey. Once submitted, questionnaires remain in the data pool in line with the anonymity of the data.

8.2. *Publication*

The results will be published in a high-ranking international journal.

9. Literature

Abadie, Gay S. The impact of presumed consent legislation on cadaveric organ donation: cross-country study. *J Health Econ* 2006;25:599–620. doi:10.1016ej.jhealeco.2006.01.003

Bastami S, Mathes O, Krones T, *et al.* Systematic review of attitudes toward donation after cardiac death among healthcare providers and the general public. *Crit Care Med* 2013;**41**:897–905. doi:10.1097eCCM.0b013e31827585fe

Bilgel F. The impact of presumed consent laws and institutions on deceased organ donation. *EurJ Health Econ HEPAC Health Econ Prei Care* 2012;13:29–38. doi:10.1007es10198-010-0277-8

Childress, J, Livermnan, CT (Ed.) (2006) *Organ Donaton: Opportunites or Acton*. doi:10.17226e11643

Christe B. Scotland plans to move to opt-out system for organ donation. *BMJ* 2017;**358**:j3298.doi:10.1136ebmj.j3298

Coppen R, Friele RD, Gevers SKM, *et al.* The impact of donor policies in Europe: a steady increase, but not everywhere. *BMC Health Seri Res* 2008;8:235. doi:10.1186e1472-6963-8-235

Coppen R, Friele RD, Gevers SKM, *et al.* Imagining the impact of different consent systems on organ donation: the decisions of next of kin. *Death Stud* 2010;34:835–47.

de Groot J, Vernooij-Dassen M, Hoedemaekers C, *et al.* Decision making by relatives about brain death organ donation: an integrative review. *Transplantation* 2012;93:1196–211. doi:10.1097eTP.0b013e318256a45f

European Commission. Eurobarometer 72.3: Public Health attitudes, Behavior, and Prevention, October 2009: Version 1. 2013. doi:10.3886eICPSR32441.v1

Federal Centre for Health Education (BZgA) (2015) Report on the 2014 Representative Survey Knowledge, Attitude and behaviour of the General Public Regarding Organ and Tissue Donation. https://www.organspende-info.de/sites/all/files/files/files/2015_12/Organ_und_Gewebes

Gimbel RW, Strosberg M , Lehrman SE, *et al.* Presumed consent and other predictors of cadaveric organ donation in Europe. *Prog Transplant* 2003;13:17–23.

Hansen SL, Eisner MI, Pfaller L, Schicktanz, S. “Are You In or are You Out?!” Moral appeals to the Public in Organ Donation Poster Campaigns: multimodal and Ethical analysis. *HealthCommunicaton,* 2017 :1–15

Haugland C, Eidsvik M. Factors influencing the relatives decision regarding organ donation in interaction with the intensive care nurses - a meta-synthesis. Published Online First: 9 May 2016.htps:eebrage.bibsys.noexmluiehandlee11250e2407227 (accessed 3 Feb 2017).

Hawkes N. Welsh opt-out law fails to increase organ donations. *BMJ* 2017;359:j5659. doi:10.1136ebmj.j5659

Healy K. Do presumed-consent laws raise organ procurement rates. *DePaul Rei* 2005;**55**:1017.

Hershenov DB, Delaney JJ. Mandatory autopsies and organ conscripton. *Kennedy Inst Ethics J* 2009;19:367–391.

Hoeyer K, Schicktanz S, Deleuran I: Public attitudes to financial incentive models for organs: a literature review suggests that it is time to shift the focus from 'financial incentives' to 'reciprocity'. Transpl Int 2013, 26(4):350–357.

Inthorn, J, Wöhlke, S, Schmidt, F, Schicktanz, S. Impact of gender and professional education on attitudes towards financial incentives for organ donation: results of a survey among 755 students of medicine and economics in Germany, 2014, BMC Medical Ethics, 15, 56, 10.1186/1472-6939-15-56

Irving MJ, Tong , JS, *et al.* Factors that influence the decision to be an organ donor: a systematic review of the qualitative literature. *Nephrol Dial Transplant Of Publ Eur Dial Transpl Assoc - Eur Ren Assoc* 2012;**27**:2526–33. doi:10.1093endtegfr683

Iacobucci G. Proposals for opt-out organ donation launched for England. *BMJ* 2017;**359**:j5764.doi:10.1136ebmj.j5764

Leins C. Should the Government decides if You're an Organ Donor? [online www.usnews.com, Feb 12, 2016. [Access date: Jan 23, 2018] .

Neto GB, Katarina Campelo , Nunes da Silva E. The impact of presumed consent law on organ donation: an empirical analysis from quantile regression for longitudinal data. *Berkeley Program Law Econ* 2007.

Nordfalk F, Olejaz M, Jensen MB, *et al.* From motivation to acceptability: a survey of public attitudes towards organ donation in Denmark. *Transplant Res* 2016;5:5. doi:10.1186es13737-016-0035-2

Nordfalk F, Olejaz M, Jensen MB, Skovgaard LL, Hoeyer K. From motivation to acceptability: a survey of public attitudes towards organ donation in Denmark. Transplant Res. 2016;5:5

Mossialos E, Costa-Font J, Rudisill C. Does organ donation legislation affect individuals' willingness to donate their own or their relative's organs? Evidence from European Union survey data. *BMC Health Seri Res* 2008;8:48. doi:10.1186e1472-6963-8-48

Organ Donation Taskforce. The potential impact of an opt out system for organ donation in the UK: an independent report from the Organ Donation Taskforce. 2008. htp:eewebarchive.natonalarchives.gov.uke/ehtp:ewww.dh.gov.ukeenePublicatonsandsta tstcsePublicatonsePublicatonsPolicy ndGuidanceeDH0090312 (30e11 e2017).

Palmer M. Opt-out systems of organ donation: International evidence review. Welsh Government Social Research 2012. http//:eegov.walesestatstcs-and-researcheopt-out-systems-oforgan-donatone?lang=en (16e12e2017)

Proposal for a Directive of the European Parliament and of the Council on standards of quality and safety of human organs intended for transplantation. Brussels: European Parliament 2008.

Radcliffe Richards, J (2012) *The ethics of transplants: Why careless thought costs lives*. Oxford University Press

Radunz S, Benkö T, Stern S, Saner FH, Paul A, Kaiser GM (2015) Medical students' education on organ donation and its evaluation during six consecutive years: results of a voluntary, anonymous educational intervention study. Eur J Med Res 20:1–5. doi:10.1186 /s40001-015-0116-6

Randhawa G, Brocklehurst , Pateman R, Kinsella S, Parry V. 'Opting-in or opting-out?'- the views of the UK's faith leaders in relation to organ donation. Health Policy. 2010 Jun;96(1):36–44

Rithalia , McDaid C, Suekarran S, *et al.* Impact of presumed consent for organ donation on donation rates: a systematic review. *BMJ* 2009;338:a3162.

Rodrıguez- Arias D, Morgan M. “Nudging” Deceased Donation Through an Opt-Out System: Libertarian approach or Manipulation? *Am J Bioeth* 2016;16:25–8. doi:10.1080e15265161.2016.1222022

Rodríguez-Arias, D.; Wilkinson, D.; Youngner, S.: How Can You Be Transparent About Labeling the Living as Dead? 2017, AJoB 17(5):24-25, 0.1080/15265161.2017.1299243

Sahin H, Abbasoglu O (2015) Attitudes of medical students from different countries about organ donation. Exp Clin Transplant.
doi:10.6002/ect.2014.0228

Samuel L. To solve organ shortage, states consider ‘opt-out’ organ donation laws. [online www.statenews.com. July 6, 2017. [access date: Jan 23, 2018] .

Schicktanz, S., Hoeyer, K. (2013): Constructing “the public” attitudes toward financial incentives for organ donation: methodological and ethical implications, In: Randhawa, G., Schicktanz, S.: Public Engagement in Organ Donation and Transplantation, Pabst Publishers, 10-17.

Schicktanz, S, Pfaller, L, Hansen, SL, Boos, M. Attitudes towards brain death and conceptions of the body in relation to willingness or reluctance to donate: results of a student survey before and after the German transplantation scandals and legal changes, Journal of Public Health, 1-8, 10.1007/s10389-017-0786-3

Schweda M, Schicktanz S (2009) The Bspare parts person^? Conceptions of the human body and their implications for public attitudes towards organ donation and organ sale. Philos Ethics Humanit Med 4:1–10. doi:10.1186/1747-5341-4-4

Shah SK, Kasper K, Miller FG. Narrative review of the empirical evidence on public attitudes on brain death and vital organ transplantation: the need for better data to inform policy. *J Med Ethics* 2015;41:291–6. doi:10.1136emedethics-2013-101930

Shepherd L, O’Carroll RE. Awareness of legislation moderates the effect of opt-out consent on organ donation intentions. *Transplantation* 2013;95:1058–63. doi:10.1097eTP.0b013e318284c13f

Shepherd L, O’Carroll RE, Ferguson E. An international comparison of deceased and living organ donation of transplant rates in opt-in and opt-out systems: a panel study. *BMC Med* 2014;12:131. doi:10.1186es12916-014-0131-4

Simpkin L, Robertson LC, Barber VS, *et al.* Modifiable factors influencing relatives’ decision to offer organ donation: systematic review. *Bmj* 2009;338:b991.

Wöhlke, S, Inthorn, J, Schicktanz, S. The Role of Body Concepts for Donation Willingness. Insights form a Survey with German Medical and Economics Students, 2016. In Jox, RJ, Assadi, G, Marckmann, G (Eds.): Organ Transplantation in Times of Donor Shortage. Challenges and Solutions, Springer, p. 27-49.

Survey questionnaire

This survey examines what European university students think about organ donation and transplantation. The survey will be undertaken in Belgium, Denmark, Germany, Greece, Israel, the Netherlands, Romania, Slovenia, Spain and England. It is the first comparative survey of organ donation and transplantation with students in Europe and we very much appreciate your participation.
The survey will take about 15-20 minutes.

First we would like to ask about your prior experiences regarding organ donation and transplantation.

1. Is there anyone in your circle of family and friends who has donated an organ after death?
Yes ☐ No ☐ Don't know ☐

2. Is there anyone in your circle of family or friends who has received an organ?
Yes ☐ No ☐ Don't know ☐

3. Is there anyone in your circle of family or friends with a serious chronic organ disease who might be in need of an organ transplant?
Yes ☐ No ☐ Don't know ☐

What do you know about the legal regulation of organ donation in your country?

4. There exist different legal systems for how citizens consent to the donation/procurement of their organs after death. Please indicate, which legal system is currently in place in your country. *(Please give* ***one*** *answer only)*

- Informed consent / opt-in system (explicit expressed wish) ☐
- Presumed consent / opt-out system (not refused during lifetime) ☐
- Other ☐ *(please state)* _____________
- I don't know ☐

5. **What do you know about the legal procedures to express one's personal choice for or against donation in your country?** *(Please respond to* ***each*** *of the following statements)*

a) In my country there are official donor cards where I can express the wish to donate or to reject organ donation.
Yes ☐ No ☐ Don't know ☐

b) In my country there is an official donor registry where I can express my wish to donate organs.
Yes ☐ No ☐ Don't know ☐

c) In my country there is an official donor registry where I can express the wish to donate or to reject organ donation.
Yes ☐ No ☐ Don't know ☐

d) In my country there is an official refusal registry where I can express my wish not to donate organs.
Yes ☐ No ☐ Don't know ☐

e) In my country, there exists no possibility to express my personal wishes about organ donation.
Yes ☐ No ☐ Don't know ☐

f) In my country I have to communicate my preferences to my family because they will be consulted about organ donation.
Yes ☐ No ☐ Don't know ☐

6. According to the law in YOUR country, when the deceased person HAD expressed a preference regarding organ donation, what is the role of the family? (Please give **one** answer only)

- The family is not consulted at all about organ donation (no role) ☐
- The family is only asked to communicate the updated preferences of the deceased in case these had recently changed ☐
- The family can veto organ donation when the deceased had consented to organ procurement ☐
- The family can authorise organ donation when the deceased had refused organ procurement ☐
- The family can both, veto and authorise organ donation ☐
- Don't know ☐

7. According to the law in YOUR country, when the deceased person HAD NOT expressed a preference regarding organ donation, what is the role of the family? (Please give **one** answer only!)

- The medical team decides alone, the family is not consulted (but the team may prevent organ
- procurement if there is evidence of family distress) ☐
- The family can oppose organ donation ☐
- The family can authorize organ donation ☐
- Don't know ☐

8. There exists in Europe different regulations governing when organs can be procured after death. What type of death is allowed for the procurement of organs in your country? (Please give **one** answer only)

- Only donation after the irreversible cessation of the functions of the brain (in some countries brain death means brain stem death) ☐
- Only donation after the irreversible cessation of circulation ☐
- Both donation after the irreversible cessation of the functions of the brain, and donation after the irreversible cessation of circulation ☐
- Don't know ☐

The following questions ask about your personal opinion towards organ donation and how it might be regulated.

9. Would you donate your organs after death?

Yes ☐ No ☐ Don't know ☐

10. Have you explicitly expressed your preference about organ donation and if so, how: *(Please respond to **each** of the following statements)*

a) By a donor card
Yes ☐ No ☐ Don't want to tell ☐
b) By a public/legal registry
Yes ☐ No ☐ Don't want to tell ☐
c) By informing my close relatives about my preferences
Yes ☐ No ☐ Don't want to tell ☐
d) By other means ☐ *(please state)* ____________

If you answered 'yes' to any of the statements in Question 10, please proceed to Question 12.

If you answered 'no' to all of the statements in Question 10, please answer question 11

11. Which of these reasons best explain why you have not expressed your wishes?
*(Please fill out **each** line)*

a) I haven't yet thought about the issue
Yes ☐ No ☐ Don't know ☐
b) I'm undecided whether I want to donate
Yes ☐ No ☐ Don't know ☐
c) I'm afraid that physicians might be more interested in my organs than in saving my live
Yes ☐ No ☐ Don't know ☐
d) I haven't had the opportunity to express my wish
Yes ☐ No ☐ Don't know ☐
e) In my country I don't need to be registered to become an organ donor
Yes ☐ No ☐ Don't know ☐
f) I don't know how to/where I can make my wish clear concerning organ donation
Yes ☐ No ☐ Don't know ☐

12. What do you know about the attitudes towards organ donation in your social environment? (Please give **one** answer only)

- My social environment is in favour of organ donation ☐
- My social environment is against organ donation ☐
- I do not know ☐

13. What do you believe are the attitudes towards organ donation among the majority of the population in your country? (Please give **one** answer only)

- The majority of the population is for organ donation ☐
- The majority of the population is against organ donation ☐
- I do not know ☐

The following questions ask your opinion about which organs or body parts should be considered for deceased donation?

14. Which organs and body parts you would like to donate after death? *(Please tick one answer in **each** line)*

Fully Reject Fully Accept

	1	2	3	4	5	6
Bone						
Cornea						
Face						
Genitalia						
Heart						
Individual limbs (e.g. a hand, arm, or leg)						
Intestine						
Kidney						
Larger area of skin						
Liver						
Lungs						
Pancreas						
Uterus						
None						

15. In many countries, organs are procured after brain death. Which of these statements do you agree with? *(Please tick one answer in **each** line)*

Fully Reject Fully Accept

	1	2	3	4	5	6
When the brain is totally and irreversibly damaged and completely stopped functioning, but the person is still on mechanical ventilation, the he/she is DEAD						
When the brain is totally and irreversibly damaged and completely stopped functioning, although the person is on mechanical ventilation, he/she is ALIVE						
When those regions of the brain connected with personality, thinking, and speaking are totally and irreversibly damaged and have completely stopped functioning, the individual is dead although he/she is on mechanical ventilation						
The individual is dead when he or she is irreversibly unconscious and will never wake up again, even if the body is kept by technical means functioning						

16. Why would you decide in favour of donating an organ after death? *(Please tick one answer in **each** line)*

a) I want to help other people
Yes ☐ No ☐ Don't know ☐
b) Responsibility to society
Yes ☐ No ☐ Don't know ☐
c) Moral sense of duty
Yes ☐ No ☐ Don't know ☐
d) Because it would get special social recognition
Yes ☐ No ☐ Don't know ☐
e) It is a medical advance that is essential
Yes ☐ No ☐ Don't know ☐
f) My body is useless after I die
Yes ☐ No ☐ Don't know ☐
g) I also want to get an organ if I need one (reciprocity)
Yes ☐ No ☐ Don't know ☐
h) Giving sense to my own death
Yes ☐ No ☐ Don't know ☐

17. Why would you potentially decide against donating an organ after death?
*(Please tick one answer in **each** line)*

a) It constitutes an invasion of my bodily integrity
Yes ☐ No ☐ Don't know ☐
b) Fear that the recipient's body will reject my organ.
Yes ☐ No ☐ Don't know ☐
c) It harms my soul's integrity
Yes ☐ No ☐ Don't know ☐
d) Lack of trust in physicians / medical care
Yes ☐ No ☐ Don't know ☐
e) Distress for my family
Yes ☐ No ☐ Don't know ☐
f) Concern that there is no proper end-of-life care
Yes ☐ No ☐ Don't know ☐
g) Fear of what happens during organ procurement
Yes ☐ No ☐ Don't know ☐
h) No right to decide to whom my organs go
Yes ☐ No ☐ Don't know ☐
i) I do not believe donors are really dead
Yes ☐ No ☐ Don't know ☐
j) None of the above ☐ *(please explain why)* ___________

The following questions are about receiving an organ from a deceased donor

18. In the case that you were in need of an organ/body parts, which of the following would you accept? *(Please tick one answer in **each** line)*

	1	2	3	4	5	6
	Fully Reject					Fully Accept
Bone						
Cornea						
Face						
Genitalia						
Heart						
Individual limbs (e.g. a hand, arm, or leg)						
Intestine						
Kidney						
Larger area of skin						
Liver						
Lungs						
Pancreas						
Uterus						
None						

19. If all of the following options were equally available and equally effective, which one would you prefer? *(Please tick one answer in **each** line)*

	Yes	No	Don't Know
The organ of a brain dead donor			
The organ of a person with no heart activity (known as non-heart beating donor. Not possible for the heart)			
An organ of an animal (e.g. pig, monkey) (xenotransplant)			
An organ grown from stem cells			
A 3D printed organ			
An artificial/mechanical organ			
I don't care as long as I recover			
I oppose any kind of organ transplantation			

The following questions concern your image of the human body.

20. Which of the following statements on the human body would you agree with? *(Please tick one answer in* ***each*** *line)*

	Yes	No	Don't Know
The human body is comparable to a machine; where all individual parts can simply be replaced			
The human body is more than the sum of its parts; thus, individual parts cannot always just be replaced			
Determine a person's individuality and uniqueness and should not be transplanted			

21. Which of the following organs is essential for a person's identity/personality according to your opinion? *(Please tick one answer in* ***each*** *line)*

	Yes	No	Don't Know
Brain			
Heart			
Kidney			
Liver			
Lung			
None of them			

Other ☐ *(please explain)* ____________

Please answer a few questions about issues discussed in public discourse and politics. We would like to know your personal views on these issues.

22. Which of the statements on organ donation and transplantation do you agree with? *(Please tick one answer in* ***each*** *line)*

Never Agree — Fully Agree

	1	2	3	4	5	6
There is a shortage of organs						
The use of brain-dead person as organ donors is morally problematic						
The use of non-heart-beating persons as organ donors is morally problematic						
The regulation concerning organ donation is morally problematic						
Organ donation can give comfort to mourning family						
The allocation of organs is unjust						
Organ donation gives death a meaning						
The allocation of organs is not transparent						
Organ donation is a citizen's duty						

23. Regardless of the legal system of consent for organ procurement in your country, which system do you prefer? *(Please tick one answer in* ***each*** *line)*

Fully Disagree — Fully Agree

	1	2	3	4	5	6
A system where I become a donor unless I have expressed a refusal (presumed consent)						
A system where I'm a donor only when I have explicitly expressed a willingness to donate (informed consent)						
A system where organs are mandatorily procured from the deceased, regardless of their preferences or those of their relatives						
A system where I'm legally required to express my preferences about organ procurement (e.g. on my driver license or on my passport)						

24. Regardless of the legal system of consent for organ procurement in your country, which role should families have when the preferences of the deceased ARE known?

(*Please give **one** answer only)*

- A system where the family is not consulted about organ procurement ☐
- A system where the family is only asked to communicate the updated
- preferences of the deceased in case these had recently changed ☐
- A system where the family can impede organ procurement when
- the deceased had consented organ procurement ☐
- A system where the family can authorize organ
- procurement when the deceased had refused organ procurement ☐
- A system where the preferences of the family are followed regardless
- of the preferences of the deceased ☐
- Don't know ☐

25. Regardless of the legal system of the country you live in, which role should families have when the preferences of the deceased ARE NOT known?

*(Please give **one** answer only)*

- A system where the family is not consulted about organ procurement. ☐
- The system at work prevails (e.g. organs are procured in presumed
- consent, and organs are not procured in explicit consent) ☐
- A system where the medical team decides (e.g. they may prevent
- organ procurement because of evidence of family distress) ☐
- A system where the family can oppose organ donation ☐
- A system where the family can authorize organ donation ☐
- Don't know ☐

26. It is discussed whether we should have a harmonized EU-Regulation ensuring the same regulation in each European country. Please indicate your level of agreement with each statement.

*(Please tick one answer in **each** line)*

Never Agree Fully Agree

	1	2		3	4	5	6
All countries should have an opt-out system							
All countries should have an opt-in system							
In all countries, the preferences of the family should always be followed in decision making related to organ donation							
In all countries, the preferences of the family should always be followed in decision making related to organ donation only in cases where there is no explicitly expressed preference by the deceased							
Each country should develop their own regulation on the consent system for deceased donation							
The public in each country should be involved in discussions about legal changes on the consent system for organ donation							

27. Do you feel sufficiently informed about the topic of organ donation?

Yes ☐ No ☐ Don't know ☐

28. According to your preferences, who should provide more information about organ donation? *(Please tick one answer in* ***each*** *line)*

a) The State (e.g. ministries, the government) at official websites
Yes ☐ No ☐ Don't know ☐
b) Transplant surgeons
Yes ☐ No ☐ Don't know ☐
c) Church/religious groups
Yes ☐ No ☐ Don't know ☐
d) Media
Yes ☐ No ☐ Don't know ☐
e) Public school
Yes ☐ No ☐ Don't know ☐
f) Patient support groups
Yes ☐ No ☐ Don't know ☐
g) Others *(please state)* ___________

We would now like to know about your degree course:

29. What is your main area of study? *(Please tick only* ***one*** *option)*

- Anthropology ☐
- Economics ☐
- Health Science ☐
- Humanities ☐
- Medicine ☐
- Nursing ☐
- Philosophy ☐
- Public Health ☐
- Social Work ☐
- Sociology ☐
- Other ☐ *(please state)* ___________

30. Which year you are studying in your current, main topic? *(Please tick* only ***one*** *option)*

- 2nd year (terms 4-6 or semesters 3 or 4) ☐
- 3rd Year (terms 7-9 or semesters 5 or 6) ☐
- 4th year (semesters 7 or 8) ☐
- 5th year (semesters 9 or 10) ☐

Finally, there are a few questions about yourself and your personal circumstances.

31. I am…

Male ☐ Female ☐ Prefer not to say ☐

32. How old are you?

Younger than 20 ☐ 20 to 24 ☐
25 to 29 ☐ 30 to 39 ☐
40 to 49 ☐ 50 to 59 ☐
60 to 69 ☐ 70 + ☐

33. Would you describe yourself as a religious person?

Spiritual ☐
Very religious ☐
Religious ☐
Somewhat religious ☐
Not religious ☐

34. If you are religious, what is your religion?

Buddhist ☐
Greek Orthodox ☐
Hindu ☐
Jewish ☐
Muslim ☐
Protestant ☐
Roman Catholic ☐
Russian Orthodox ☐
Sikh ☐
None ☐
Other ☐*(please state)* ___________

35. I found understanding this questionnaire…

Very easy ☐
Easy ☐
Difficult ☐
Impossible ☐

36. I found the topic of the questionnaire…

Very interesting ☐
Interesting ☐
Not very interesting ☐
Not interesting ☐

Thank you for completing the questionnaire!

If you want to take part in the raffle, please click to leave your contact data here. An e-mail address will do. The contact information will be stored and processed separately. For this purpose, you will be redirected to another website so it is not possible to trace back a single person.

Participant Information Sheet

Information about voluntary participation in student survey about Knowledge and attitudes about donation and transplantation of organs/body parts (ISSATO)

Dear participant,

Thank you for your interest in our study. We would like to invite you to take part in our research study to see what people think about the donation and transplantation of organs and body parts. Before you decide whether you would like to take part it is important for you to understand why the research is being done and what it would involve for you.

What is the purpose of the study?

Several European countries recently changed or considered to change their models of organ procurement and systems of consent for organ donation.

This is an international quantitative survey of students from different fields of health sciences and social sciences/humanities to investigate their knowledge and attitudes towards different models of consent for post-mortem organ donation in each country.

The purpose of this study is to explore the perspectives and opinions of students about these developments and to increase the understanding of the social and ethical issues involved.

Who can take part?

Anyone who is 18 years old or older and in his or her second year onwards study year at the university and study health sciences and social sciences/humanities is able to take part to our survey.

Do I have to take part?

No. It is up to you to decide if you want to take part. If you do decide to take part you will be free to withdraw from the study at any time, without giving a reason.

What will happen to me if I take part?

If you agree to take part in the study you will be asked at the end of this information shed to click to a box to start with the survey. The survey will take approximately 20 minutes.

The survey is collecting all data anonymously

What are the possible benefits of taking part?

As part of this study, you can win one of a 4 coupons up to 25 euros. If you would like to participate to the raffle, you can enter your e-mail address in the address field provided after completing the survey. The raffle will take place after completion of the survey. The personal data collect for this raffle will be collected separately from the survey data.

Your contribution to the survey will allow the researchers to have a better understanding of public perceptions of the social and ethical issues associated with recent developments in organ transplantation, and may inform policy making in this area.

Will my taking part in this study be kept confidential?

Yes. Any information you provide will be kept confidential. The survey data will be kept securely at the German University data management (GWDG) company. The survey data cannot be link to your email-address in case you take part in the raffle. All emails will be destroyed after the raffle has taken place (approx. 3 month after end of data collection)

By clicking the box, you agree with the conditions outlined here and start the survey. You can print and keep this sheet of information for your records.

Recruitment process

Participants were recruited at: Medical University Innsbruck and Private University for Health Science, Medical Informatics and Technology (Austria), University of Antwerp (Belgium), University of Copenhagen (Denmark), University of Göttingen (Germany), “Alexander” Technological Educational Institute (Greece), Alexandru Ioan Cuza University (Romania), University of Ljubljana (Slovenia), and University of Granada (Spain).

The recruitment process took place between October 2018 and November 2019 using convenience sampling methods: a link to the online survey was distributed via university mailing lists and flyers displayed on the campuses of the aforementioned universities. Student participants in Germany, Greece, Denmark, Slovenia, and Spain were offered participation in a 50€ e-commerce voucher lottery. At each participating site, partners were asked to recruit at least 100 students of Health Sciences/Medicine and 100 students of Social Sciences/Humanities.

In order to participate, students had to read and acknowledge the study information telling them that by proceeding to the questionnaire and submitting it, they consented to participate and processing the data for scientific purposes. The survey started only after they agreed to the terms. Additional information about the survey and the research project was provided via the project website (issatosurvey.wordpress.com) and linked to the online questionnaire.

This study was carried out in accordance with the European Charter of Fundamental Rights and with the Declaration of Helsinki and its later amendments. The authors obtained the required permits and approvals and they ensured that the study complies with local ethical and legal requirements for all countries involved. The study protocol was reviewed and approved as minimal risk research by the University Medical Center Göttingen Human Research Review Committee (Ref. no.: 13/01/19), as well as by the Universidad de Granada Ethics Committee on Human Research (ref. no. 718/CEIH/2018). No extra ethics review was required for this type of study in the other countries involved (Austria, Belgium, Denmark, Greece, Romania, and Slovenia). Written informed consent was obtained from all individual participants prior to participation. A blank example of the form used to inform participants is included in the Supporting Information file.

Sample composition

The sample consisted of 2,193 university students from the second year and later in eight countries: Austria, Belgium, Denmark, Germany, Greece, Romania, Slovenia, and Spain. The main demographic data as well as the consent policy in force in each country are summarised in Table 1 and Table 2. Approximately half of respondents studied health sciences and medicine, and the remaining half were students of a broad range of humanities and social science disciplines (see Rodríguez-Arias et al., 2021). A majority of respondents (76%) were women, which partially reflects an over-representation of women in the disciplines concerned (EUROSTAT, n.d.) but may also account for an answer bias.

TABLE 1. SOCIO-DEMOGRAPHIC DATA OF THE SAMPLE POPULATION IN EACH COUNTRY.

Consent policy	Country	N	Gender (F/M/ND)[a]	Health studies[b]	Aged 20-24	2nd-3rd year of study	Non-religious[c]
Opt-in	Denmark	230	166/63/1	51 %	72 %	61 %	55 %
	Germany	424	318/94/13	53 %	65 %	48 %	40 %
	Romania	190	165/24/1	29 %	74 %	91 %	14 %
Opt-out	Austria	339	252/79/8	69 %	49 %	62 %	77 %
	Belgium	439	332/104/3	18 %	87 %	54 %	64 %
	Greece	159	124/33/2	79 %	63 %	40 %	13 %
	Slovenia	190	136/50/4	54 %	89 %	82 %	31 %
	Spain	222	163/59/3	55 %	75 %	43 %	62 %

[a] Female (F)/male (M)/none declared (ND).
[b] Health-related disciplines include: health sciences, medicine, nursing, public health. Non-health related disciplines include: anthropology, economics, humanities, philosophy, social science, sociology, and social work.
[c] Participants describing themselves as non- or only slightly religious.

TABLE 2. SAMPLING FRAME. (OVERVIEW OF STUDENTS WHO MAY HAVE RECEIVED INFORMATION ABOUT THE SURVEY)

Country and City of data collection	Year	Number of students who had potential access to the survey. Medicine and Health Sciences	% of women	Number of students who had potential access to the survey. Humanities/Social Sciences, Social Work/Social Management	% of women
Austria (Innsbruck & Hall in Tirol)[3]	2019	2026 (MUI)<br>1700 (UMIT)	54 % (MUI)<br>54 % (UMIT)	4377 (LFUI)<br>260 (MCI)	52 % (LFUI)<br>52 % (MCI)
Belgium (Antwerp & Ghent)[4]	2019	2009	61%	5081	65%
Denmark (Copenhagen)[5]	2018-2019	402	72%	405	57%
Germany (Göttingen)[6]	2018-2019	3741	65%	8411	62%
Greece (Thessaloniki)[7]	2018-2019	1680	61%	610 (IHU)	39%
Romania (Babes-Bolyai, Cluj)	2018-2019	N/A	N/A	N/A	N/A
Slovenia (Ljubljana)	2018-2019	N/A	N/A	N/A	N/A
Spain (Granada)[8]	2018-2019	1349	70%	2569	57%

3 Austria: recruitment via university and faculty mailing lists, as well as promotion in courses by lecturers, at Medical University Innsbruck (MUI), the health & life sciences university Hall in Tirol (UMIT), University of Innsbruck (LFUI), and Management Center Innsbruck (MCI).

4 Belgium: recruitment via faculty mailing lists, public poster campaigns, and student involvement in specific courses.

5 Denmark: Recruitment via promotion in courses by Jensen, followed by emails with survey link to students via online course platforms

6 Germany: Recruitment via faculty mailing-lists, public poster campaigns, flyer, newsletter.

7 Greece: Recruitment via flyers and promotion in courses by lecturers, at Aristotle University of Thessaloniki (AUTH: medicine) and International Hellenic University (IHU: nursing; and also non-medical or non-health sciences students).

8 Spain: Recruitment via faculty mailing-lists, public poster campaigns, University of Granada website, as well as promotion in courses by lecturers.

Additional results on consent policies for deceased organ donation

Results from this survey on knowledge and attitudes towards consent system (not including Romania) have been used in a recent article to support a theoretical tool for assessing governance of national policies on organ procurement (Rodríguez-Arias et al., 2021). Here we present some additional analyses including data from Romanian respondents.

Awareness of national consent policies

Participants were asked which model of consent is in place in their country. We found a wide variation in correct answer rates across countries. As shown in Fig. 1, a large majority of Danish and German respondents correctly reported that opt-in and, correspondingly, most Austrian and Belgian respondents correctly indicated that opt-out is in place in their country. A majority of Romanian respondents correctly ticked the opt-in policy while about one third did not know which consent model is in place. Conversely, most participants in Spain, Greece, and especially Slovenia, wrongly believed that an opt-in policy is in place or declared that they don't know.

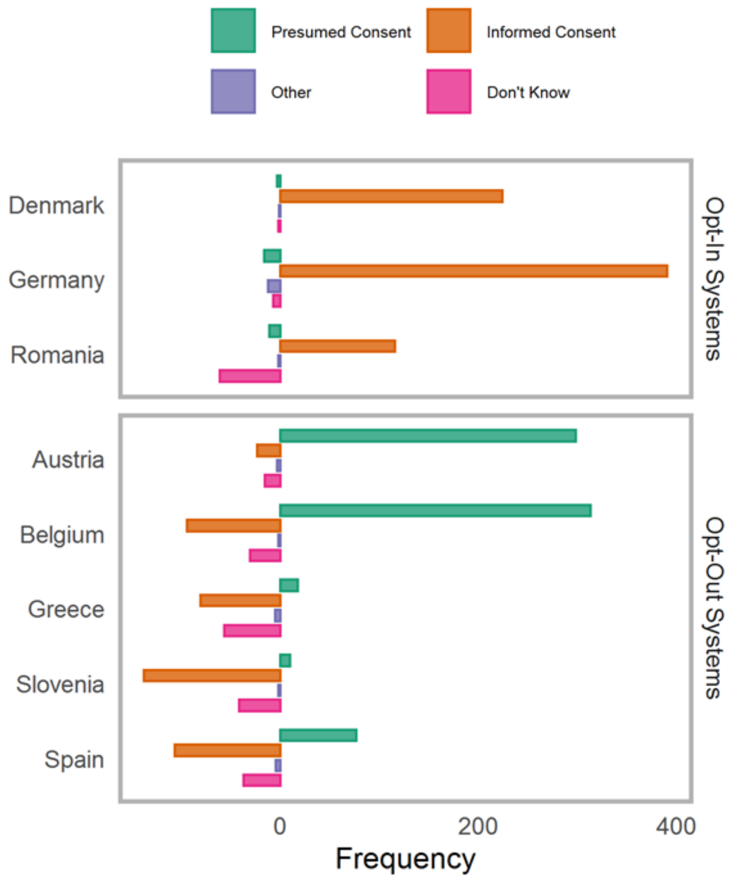

Fig. 1. Frequency bar graph of knowledge about extant consent policy by country. Note: Wrong answers are displayed as negative counts.

We were also interested in sociodemographic predictors of knowledge. Neither gender ($z = -0.67$, $p = .51$) nor religiosity ($z = -1.33$, $p = .18$) had significant effects on knowledge of the applicable consent system. Study field, however, influenced knowledge about the consent system in force as it was higher among students of medicine and health-related disciplines than among those who studied humanities and social sciences ($z = 4.45$). This was also confirmed by multiple regression analyses controlling for gender, nationality, and religiosity ($z = 4.61$, both $ps < .001$).

Preferences regarding consent policies

We then asked participants which form of consent they felt was most appropriate irrespective of the regulation in force in their country. The options to choose from were presumed consent (opt-out), informed consent (opt-in), mandatory choice (also termed mandated choice, i.e., a system in which all are legally obligated to express their preferences regarding donation), or conscription (i.e., obligatory cadaveric organ removal regardless of any preferences the deceased had expressed and their families might have).

As shown in Fig. 2, in most countries participants favoured presumed consent (paired t-tests, all $ps < .001$) and mandatory choice but clearly rejected conscription, irrespective of the consent system in place. Interestingly, respondents in Austria and Belgium (both having an opt-out system) disapproved more expressly of informed consent than those in Denmark and Germany (opt-in system). Moreover, in all these countries approval of presumed consent and mandatory choice was reflected in disapproval of informed consent. In Spain, only very few respondents expressed favour or disapproval of informed consent, and apparently considered mostly presumed consent and mandatory choice as preferable. Irrespective of these subtleties, participants from Denmark, Germany, Austria, Belgium, and Spain had a clear preference for presumed over informed consent. Remarkably, we observed a reversed pattern in Greece, Romania, and Slovenia. While presumed consent was not rejected by respondents from these countries, they had a clear preference for informed consent as well as mandatory choice.

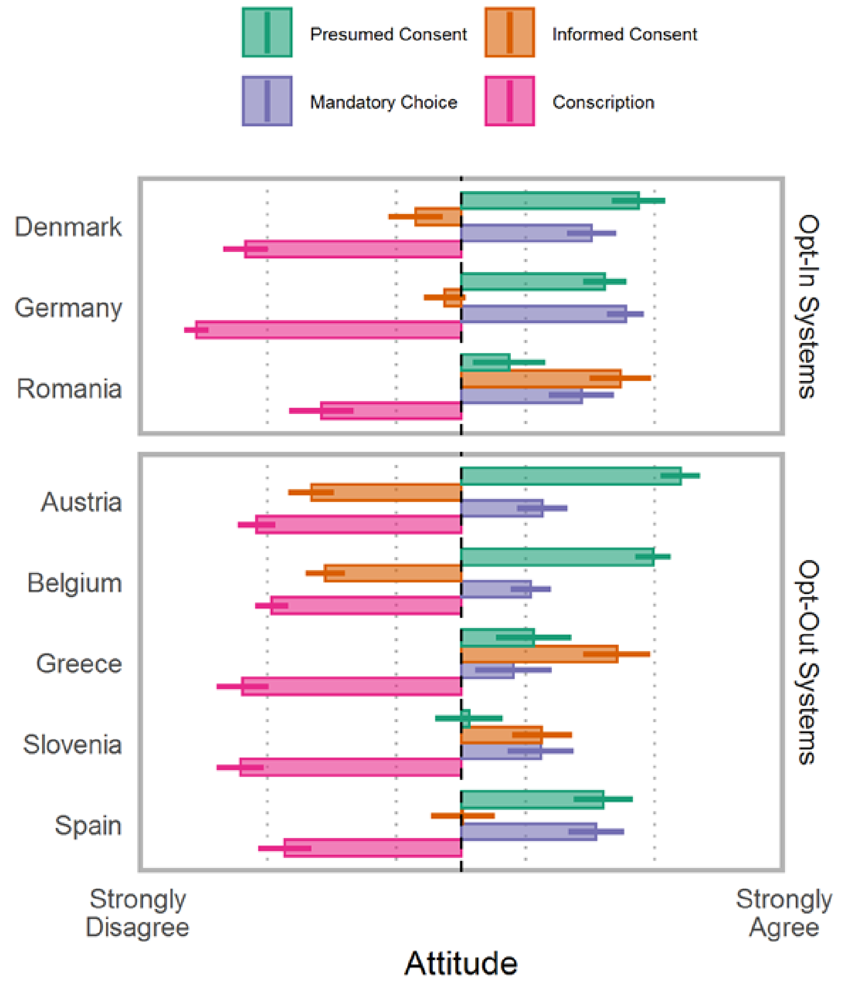

Fig. 2. Mean attitude toward each consent policy by country. A dashed vertical line represents the scale midpoint, therefore leftward bars indicate disapproval and rightward bars indicate approval. Whiskers on each bar display the 95% confidence interval.

As these findings indicated significant cultural differences with regard to preferences, we looked at differences among sociodemographic groups, in two separate ANCOVAs. After controlling for both national difference and national variation, we found that religiosity and health-related studies –but not gender– influenced attitudes towards presumed consent (see Table 3A). Moreover, students of health-related disciplines reported more favourable views about presumed consent and more disapproving views about informed consent (see Table 3B). The effect of religiosity emerged in the opposite direction: specifically, religious participants were more opposed towards presumed consent and more in favour of informed consent.

Next, we assessed how satisfied participants were with the consent system in place in their respective countries. A series of one-sample tests against the point of neutrality (mu = 3.5) revealed that respondents in Austria ** ($M = 5.20$, $p < .001$), Belgium ** ($M = 4.98$, $p < .001$), Spain ** ($M = 4.63$, $p < .001$), and Greece ** ($M = 4.03$, $p < .001$) are all predominantly satisfied with the system in place. On the contrary, in Slovenia ($M = 3.56$, $p = .67$) and Germany ($M = 3.38$, $p = .13$) they were ambivalent and in Denmark * ($M = 3.15$, $p = .001$) primarily dissatisfied.

Table 3: Effects of sociodemographic parameters on (A) attitudes towards presumed consent across and within countries and (B) on favouring/disapproving view on presumed or informed consent (results from ANCOVA analyses)

A

Sociodemographic variable	National difference	National variation
None	$F(6, 1846) = 23.5$	$F(6, 1846) = 57.9$
Religiosity	$F(1, 1846) = 58.9$	$F(1, 1846) = 34.5$
Health-related studies	$F(1, 1846) = 12.5$, $ps < .001$	$F(1, 1846) = 12.0$, $ps < .001$
Gender	$F(2, 1846) = 0.96$, $p = .38$	$F(2, 1846) = 0.96$, $p = .38$

B

Sociodemographic variable	View[i] on presumed consent	View on informed consent
Medicine & Health studies	$B = 0.28$, $t = 3.52$, $ps < .001$	$B = -0.29$, $t = 3.52$, $ps < .001$
Religiosity	$B = -0.38$, $t = 7.93$, $ps < .001$	$B = 0.30$, $t = 6.12$, $ps < .001$
